# The MFα signal sequence in yeast-based protein secretion: challenges and innovations’

**DOI:** 10.1007/s00253-025-13532-z

**Published:** 2025-06-05

**Authors:** Magdalena Merkaš, Nina Grujicic, Martina Geier, Anton Glieder, Anita Emmerstorfer-Augustin

**Affiliations:** 1 Institute of Molecular Biotechnology, Graz University of Technology, NAWI Graz, Graz, Austria; 2 Bisy GmbH, Hofstaetten/Raab, Austria; 3 Austrian Centre of Industrial Biotechnology, Acib GmbH, Graz, Austria; 4 BioTechMed-Graz, Graz, Austria

**Keywords:** Yeast, Protein secretion, Mating factor alpha, Signal sequence

## Abstract

**Abstract:**

Protein secretion in yeast is a complex, multistep process heavily reliant on signal sequences to guide recombinant proteins through the secretory pathway. Among these, the mating factor alpha (MFα) signal sequence from *Saccharomyces cerevisiae* has emerged as a powerful tool for enhancing the extracellular production of heterologous proteins. This review provides a comprehensive overview of the MFα signal sequence, tracing its historical development and role in advancing our understanding of protein secretion mechanisms, including co- and post-translational secretory pathways. We highlight key studies focused on optimizing the MFα signal sequence for improved secretion efficiency, leading to the development of several highly effective variants. These optimized sequences have significantly increased recombinant protein yield and quality, with notable implications for both research and industrial applications. Additionally, we explore the challenges of MFα-based secretion, including issues of missorting, incorrect processing, and aggregation in the endoplasmic reticulum (ER). We discuss emerging strategies to overcome these bottlenecks, such as fusion with alternative signal sequences and strain engineering. Finally, the review highlights current efforts to develop more robust signal peptides, and underscores the importance of continued innovation in protein secretion systems to meet the growing demand for high-quality recombinant proteins in biotechnological and therapeutic applications.

**Key points:**

• *MFα remains the top choice for recombinant protein secretion in yeast*

• *Challenges in secretion: ER aggregation, missorting, and processing errors*

• *Mutated and hybrid signal peptides offer promising solutions*

## Introduction

Secretory protein production is a highly efficient strategy for recombinant protein expression, as it simplifies downstream processing and reduces overall production costs. This approach relies on a signal sequence—a short peptide fused to the protein’s N-terminus—that directs the nascent protein into the secretory pathway, ensuring its proper translocation across cellular membranes. Although various signal sequences are available, only a few are well studied. One of the most extensively studied is the MFα signal sequence in yeast. Originally identified for its role in the *Saccharomyces cerevisiae* mating pathway, the MFα signal sequence directs the MFα pro-pro-hormone into the secretory pathway, enabling the secretion of α-factor (Kurjan and Herskowitz 1982). Research on MFα has not only elucidated its importance for yeast mating but has also provided key insights into eukaryotic protein secretion, signal transduction, and cellular communication. Beyond its mating role, the MFα signal sequence has been widely adopted to facilitate heterologous protein secretion in yeast. By fusing MFα to a target protein’s N-terminus, researchers have directed proteins into the yeast secretory pathway for extracellular production. This approach was first demonstrated by Brake et al., who successfully secreted epidermal growth factor in *S. cerevisiae* (Brake et al. 1984). While traveling through the secretory pathway, the MFα signal sequence undergoes processing in three steps: recognition by the signal recognition particle and translocation across the endoplasmic reticulum, cleavage and N-glycosylation in the pro-region, and final processing in the Golgi by Kex2 endopeptidase and Ste13 dipeptidyl aminopeptidase (Julius et al. 1983; Johnson et al. 2013; Aggarwal and Mishra 2020). The processing of the MFα secretion signal sequence is not limited to its native organism, *Saccharomyces cerevisiae*, nor to its native protein, mating factor α peptide. Thus, recombinant protein secretion using the MFα signal sequence has been successfully achieved in various yeast species, including *S. cerevisiae* (Brake et al. 1984), *K. phaffii* (Clare et al. 1991), *Kluyveromyces lactis* (Jie et al. 1992), *Ogataea minuta* (Akeboshi et al. 2007), and *Hansenula polymorpha* (Weydemann et al. 1995). These findings demonstrate that recombinant secretion signal sequences can be processed across different yeast species, indicating the presence of a conserved protein secretion pathway. Key factors involved in this process, such as Kex2 and DPAPase A (Ste13), are broadly conserved, underscoring their fundamental role in yeast protein secretion.

*K. phaffii* is particularly famous for its efficient protein secretion machinery, making it one of the most prominent hosts for the secretory production of recombinant proteins. In the 1980 s, initial studies in *K. phaffii* focused on the secretory production of recombinant proteins using their native signal sequences, as demonstrated with invertase (Tschopp et al. 1987). However, the discovery that the MFα signal sequence could also be applied in *K. phaffii*, as first demonstrated by the secretory production of epidermal growth factor, led to a real breakthrough (Siegel et al. 1990; Clare et al. 1991). Even though many different signal sequences have been tested to date, including α-amylase, *SUC2*, *PHO1*, and *EXG1* signal sequences (reviewed by (Gomes et al. 2018)), the *S. cerevisiae* MFα signal peptide remains the most used leader sequence in *K. phaffii*. This is because few alternative signal peptides have consistently achieved higher product quality or higher secreted titers of recombinant protein (Neiers et al. 2021). Also, over the years, diverse modifications have led to improved variants of the MFα signal peptide, like deletion of specific amino acids (Lin-Cereghino et al. 2013), codon context (CC) optimization (Xiong et al. 2005), or mutational variations (Rakestraw et al. 2009; De Salas et al. 2019). Additionally, commercially available kits (e.g., *Pichia* Expression Kit, Invitrogen), ready-to-use plasmids (e.g., pPpT4_Alpha_S, (Näätsaari et al. 2012)), and straightforward protocols (Merkaš et al. 2025) facilitate the easy cloning and expression of recombinant genes. In this review, we provide a comprehensive overview of the MFα signal sequence with a focus on its use in *K. phaffii* for recombinant protein secretion. We summarize its processing, recent engineering advances, and practical considerations for its application. By discussing both the strengths and limitations of MFα, we aim to support researchers in selecting optimal secretion signals and engineering strategies for their expression systems.

## Protein secretion: co-translational and post-translational pathways

**Fig. 1:**
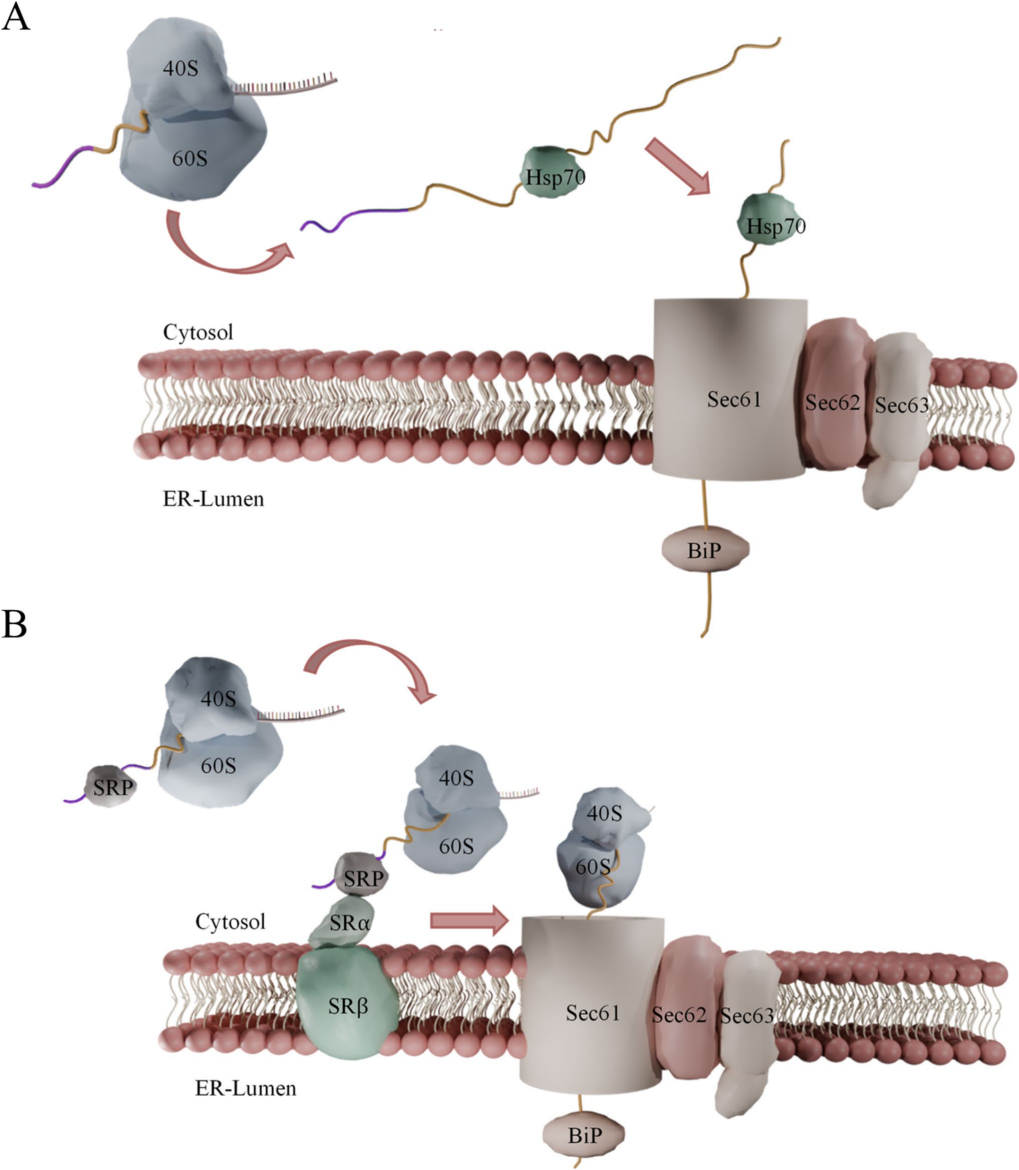
Co- and post-translational allocation of secreted proteins to the endoplasmic reticulum (ER). **A** In the post-translational pathway, cytosolic chaperones stabilize the fully synthesized polypeptide, independent of its signal sequence (shown in purple). After translation is complete, the polypeptide is directed to the heptameric Sec complex, composed of Sec61p, Sbh1, Sss1, Sec62, Sec63, Sec71, and Sec72. Translocation of the unfolded protein into the ER is facilitated by the ER-resident chaperone BiP/Kar2. **B** In the co-translational pathway, protein synthesis initiates at the ribosome, where the cytosolic α-subunit of the signal recognition particle (SRP) recognizes the N-terminal signal sequence (highlighted in green). The SRP directs the ribosome–nascent chain complex to the Sec61p translocon, enabling direct insertion of the growing polypeptide into the ER lumen. An additional co-translational translocon known in yeast is Ssh1

Protein secretion begins on ribosomes. After synthesis, signal recognition particles (SRP) recognize and bind the hydrophobic core of signal sequences and direct nascent proteins to the endoplasmic reticulum (ER) (Ng et al. 1996), where they are translocated and undergo initial processing. Vesicular transport then shuttles these proteins to the Golgi apparatus for further modification and sorting. Finally, secretory vesicles carry the proteins to the cell membrane for secretion into the extracellular environment (Delic et al. 2013; Sun and Brodsky 2019). In the 1990 s, the Walter lab found that only a subset of signal sequences require the function of SRPs for being secreted, suggesting that an alternative, SRP-independent pathway must exist (Hann and Walter 1992). These two routes were later defined as co-translational (SRP-dependent) and post-translational (SRP-independent) secretion. The choice between them is largely determined by the hydrophobicity of the signal sequence, which governs SRP recognition (Ng et al. 1996). Co-translationally synthesized proteins are directed to the ER during translation, while post-translationally synthesized proteins are selectively targeted for translocation into the secretory pathway after translation has been completed (reviewed by Delic et al. (2013)). Both processes involve the Sec61 complex but utilize different channel partners (Fig. 1). Once translocated to the ER lumen, the signal peptide is cleaved off (Gierasch and Kaiser 1989), allowing properly folded and modified proteins to proceed to the Golgi apparatus. In the Golgi, proteins undergo final post-translational modifications, including the removal of any pro-peptide if present. The proteins are then packaged into vesicles and transported to their cellular destination, typically the plasma membrane, where vesicles fuse with the membrane to release their contents into the extracellular space (Raschmanová et al. 2021). In rare cases, alternative protein secretion pathways that bypass the traditional ER-Golgi route have been reported, as for example shown for galectin-3 (Mehul and Hughes 1997) and thioredoxin (Matsuo et al. 2001) in mammalian cells. These proteins do not display any signal sequence or protein motif known to act as a signal for export (reviewed by Kuchler et al. (1997)).

### The MFα signal sequence and the post-translational secretory pathway

The MFα signal sequence directs secreted proteins into the post-translational secretory pathway (Ng et al. 1996). In this pathway, nascent proteins destined for secretion undergo post-translational (ribosome-uncoupled) translocation into the endoplasmic reticulum (ER) lumen, a process influenced by the hydrophobicity and amino acid composition of the already fully translated signal peptide (Zimmermann et al. 2011). Once polypeptides are released from the ribosome, they need to remain in an unfolded or loosely folded state and be protected from aggregation before translocation. This unstructured state is stabilized by cytosolic chaperone complexes, such as those of the Hsp70 family, through interactions that are independent of the secretion signal sequence (Plath et al. 1998). In *S. cerevisiae*, cytosolic chaperones such as Ssa1 and Ydj1 help maintain the unfolded state of the protein and dissociate just before translocation into the ER occurs (Plath and Rapoport 2000) (Fig. 1A). Transport into the ER is facilitated by the heptameric SEC complex, which comprises of the Sec61 translocon pore, Sbh1, Sss1, Sec62, Sec63, Sec71, and Sec72 (Fig. 1A) (Denks et al. 2014; Loibl et al. 2014; Barbieri et al. 2023). Instead of the SRP, Sec61, Sec62, and Sec72 recognize and bind to the signal peptide, e.g., the MFα signal sequence, guiding the nascent protein into the ER (Plath et al. 2004). It is believed that posttranslational translocation into the ER operates via a Brownian ratchet mechanism, where the polypeptide chain moves back and forth within the translocon pore and is then captured in the ER-lumen through the binding of the ER chaperone BiP/Kar2, a member of the Hsp70 chaperone family (Matlack et al. 1999)(Simons et al. 1995; Pobre et al. 2019). Once in the ER, the proteins finally undergo critical processes such as folding, disulfide bond formation, and the initiation of post-translational modifications, including glycosylation (Puxbaum et al. 2015). It was also reported that the process of posttranslational translocation can be slowed down or even stop if parts of the secreted protein fold prematurely in the cytosol (Schatz and Dobberstein 1996). A well-studied example is GFP, where initial folding of immature msGFP showed to impede translocation to a major extend, resulting of missorting of the protein to the vacuole (Fitzgerald and Glick 2014).

### The less well-studied co-translational secretory pathway

Due to the historical focus on simpler post-translational mechanisms and the technical challenges associated with analyzing the integrated processes of simultaneous translation and translocation, co-translational protein secretion has been studied less extensively than post-translational secretion. During co-translational secretion, the nascent protein and the ribosome have to interact with the SRP, the signal recognition receptor (SR), and one of the translocon pores, Ssh1 or Sec61 (Fig. 1B). The SRP is a multi-protein complex composed of Srp14, Srp21, Srp54, Srp68, Srp72, and Sec65, along with 7S single RNA (SCR1) (Fitzgerald-Hayes et al. 1982). Functionally, the SRP recognizes and binds the signal sequence of the nascent protein emerging from the ribosome. This ribosome-protein-SRP complex subsequently engages with the cytosolic moiety of the signal recognition receptor, SRα, and initiates the GTP dependent translocation (Miller et al. 1995). As for the post-translation translocation, the transport of the nascent protein chain into the ER is mediated via BiP/Kar2 through the translocon pore Sec61 (Pool 2022).

## Signal peptides

Signal peptide sequences, though not highly conserved, share common features across proteins. Typically 25–30 amino acids long, they exhibit a tripartite structure. The N-terminal segment (N-region) consists of 1–5 positively charged residues that enhance interactions with negatively charged elements, such as the phosphate head groups of lipids in the ER membrane and the SRP complex. These interactions are essential for effective protein targeting and translocation into the endoplasmic reticulum (Owji et al. 2018). Following the N-terminal region, signal peptides contain a hydrophobic core, known as the H-region. The H-region contains around 8 hydrophobic residues, supporting peptide conformation and orientation toward the membrane. The H-region also plays a critical role in the cleavage of the signal peptide, facilitating the protein’s progression through the secretory pathway. Additionally, it is instrumental in post-translational modifications, such as N-linked glycosylation, which are essential for the protein’s functionality and stability (Owji et al. 2018). The C-terminal segment, or C-region, features a polar domain of 3–7 residues that aids in directing the peptide to its destination (Von Heijne 1990; Owji et al. 2018). The C-terminal region is typically rich in glycine and proline residues, which disrupt alpha-helical structures, as well as amino acids with short side chains at the −1 and −2 positions. This region includes residues recognized by a signal peptidase for cleavage, ensuring efficient processing and release into the secretory pathway. Proper amino acid composition in these regions is crucial for efficient cleavage and protein maturation (Owji et al. 2018; Von Heijne 1984; Robinson and Bulleid 2020).

Some signal peptides, such as the MFα signal peptide, consist of pre- and pro-regions (see Chapter 3.1 for more detail), whereas others, like the Ost1 signal sequence, contain only pre-sequences (Barrero et al. 2018). Although many signal peptides have been identified, the role and necessity of pro-regions are not fully understood. Signal sequences containing only a pre-region direct proteins into the secretory pathway, where the signal peptide is cleaved after translocation. In contrast, prepro regions include an additional pro-sequence that can assist with protein stabilization, correct folding, or proteolytic activation of the mature protein (Baker et al. 1993). Whether the presence of a pre or prepro region determines co-translational versus post-translational translocation into the endoplasmic reticulum (ER) remains unclear. What is known, however, is that the signal sequence itself, rather than the mature protein, determines the specificity of the secretion pathway (Feldheim and Schekman 1994). This aspect of protein translocation remains an area of active research, as understanding these mechanisms could provide insights into protein sorting and processing within cells.

To recognize sorting signals in prokaryotic and eukaryotic cells, numerous tools have been developed, focusing on distinguishing signal peptides from non-secretory proteins and predicting cleavage sites within signal peptides. Besides experimental methods, computational tools help analyze if a protein contains an N-terminal signal sequence. In eukaryotes, these tools use biological or empirical sequence features for protein localization, employing machine learning algorithms like k-nearest neighbors, random forest, support vector machines (SVM), and deep learning (Imai and Nakai 2020). SignalP, a key tool since 1996, uses neural networks to identify positions within signal peptides and detect cleavage sites (Nielsen et al. 2019). Its latest version, SignalP 6.0 (2022), leverages protein language models trained on extensive datasets, capturing biological characteristics and structure. While SignalP primarily predicts classical signal peptides, it does not distinguish pre- and pro-regions within these peptides (Teufel et al. 2022). For complex cases, such as the MFα signal peptide (with both pre- and pro-regions) or Ost1 (pre-sequence only), additional tools or experimental methods, like sequence alignment or proteomic analysis, are needed to characterize pro-regions.

Importantly, not all secreted proteins follow the classical secretion route through the ER, Golgi apparatus, and plasma membrane into the extracellular space. Alternative mechanisms, collectively referred to as Unconventional Protein Secretion (UPS), are regulated by signaling processes that do not necessarily involve pre-pro-sequences. The complexity and diversity of UPS pathways have been extensively reviewed in recent years (Nickel and Seedorf 2008; Nickel and Rabouille 2009; Rabouille 2017; Cohen et al. 2020).

### Processing and structural features of the MFα signal sequence

**Fig. 2:**
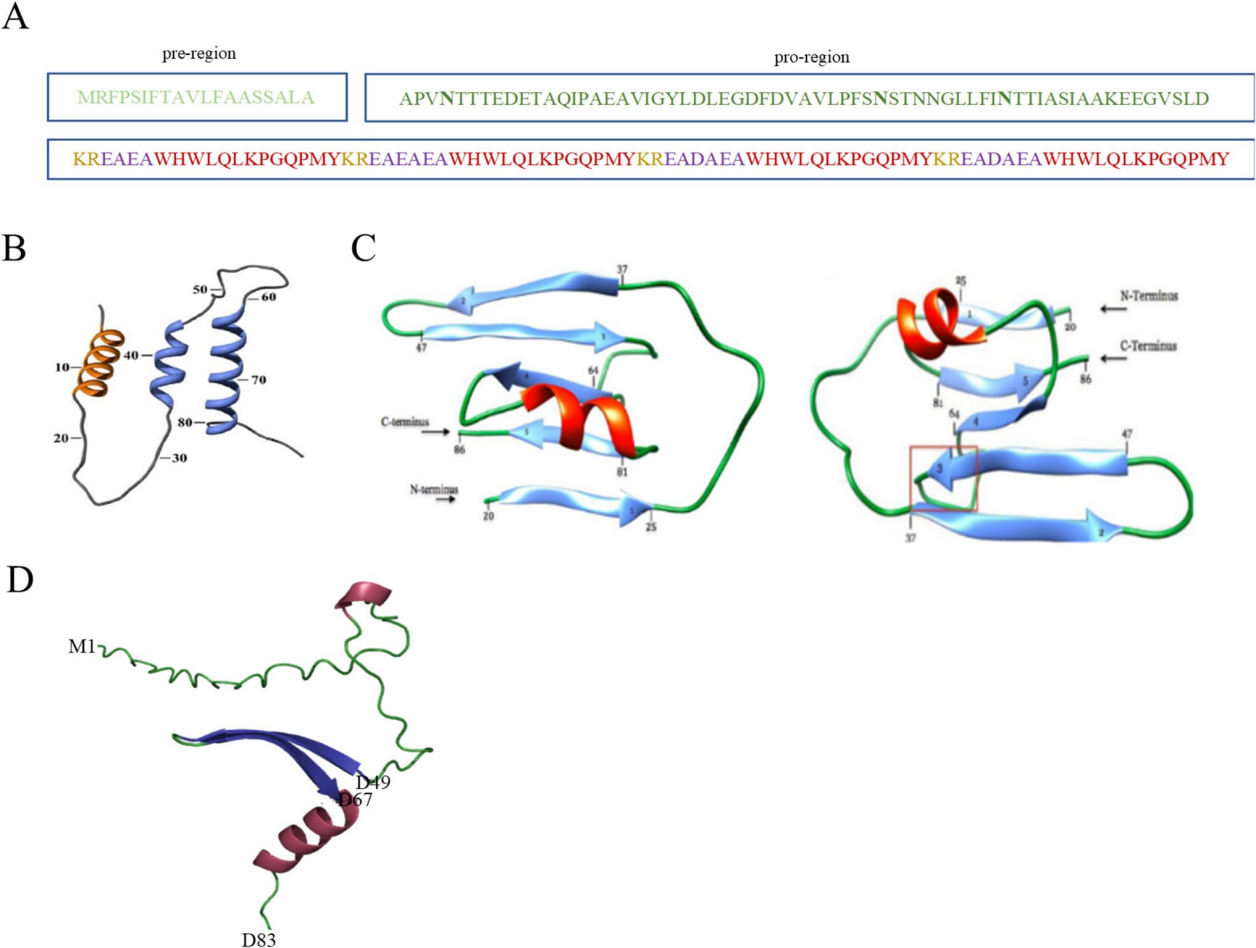
*Saccharomyces cerevisiae* mating factor α peptide sequence and predicted structures of the MFα prepro regions. **A** The α-factor peptides are synthesized as 165-amino-acid prepro-proteins. The secretory signal sequence begins with the pre-region, which directs post-translational translocation into the endoplasmic reticulum (ER). The pro-region undergoes proper folding and N-glycosylation (bold N-sites), facilitating further transit through the secretory pathway. Processing includes cleavage by the Kex2 protease at the dibasic KR sequence (yellow) and removal of EAEA or DEAE (purple) repeats by DPAPase A in the Golgi. The mature 13-amino-acid MFα peptides (red) are then secreted. **B** Predicted tertiary structure of the MFα prepro-leader sequence, with the pre-region (amino acids 1–19) shown as an orange α-helix, and two predicted α-helices in the pro-region, shown in blue (Chahal et al. 2017). **C** Structure models based on circular dichroism (CD) data (Chahal et al. 2017). **D** AlphaFold3 prediction of the prepro-leader sequence (Abramson et al. 2024)

The *Saccharomyces cerevisiae* MFα signal sequence and mating factor α peptides are synthesized as 165-amino-acid prepro-proteins (UniProt P01149). The pre- and pro-regions of the signal sequence are followed by a tandem of four α-factor peptides (Fig. 2A) (Kurjan and Herskowitz 1982). After post-translational targeting to the ER membrane, the 19-amino-acid pre-region is cleaved off by unidentified ER membrane-bound peptidases (Bar-Nun et al. 1980; Lively and Walsh 1983). The MFα protein is then folded correctly in the ER lumen (Ellgaard and Helenius 2003) and undergoes post-translational modifications, including N-glycosylation of three sites on the 64-amino-acid MFα pro-region (Fig. 2A) (Julius et al. 1984; Caplan et al. 1991). As it progresses along the secretory pathway, the MFα pro-protein undergoes several endoproteolytic cleavages, ultimately producing the secreted 13-amino-acid MFα peptides. The first cleavage, by the Ca^2^⁺-dependent serine protease Kex2 in the late Golgi (Fuller et al. 1989; Redding et al. 1991) occurs at the dibasic Lys-Arg (KR) repeats (Fig. 2A) (Julius et al. 1984). The final processing step involves the removal of N-terminal Glu-Ala-Glu-Ala (EAEA) or Asp-Ala-Glu-Ala (DAEA) repeats by the membranous dipeptidyl aminopeptidase DPAPase A (Ste13) (Julius et al. 1983; Voos and Stevens 1998).

The Lin-Cereghino lab provided interesting insights into the structure–function relationship of the MFα prepro leader peptide (Chahal et al. 2017). The initial model generated by the Jpred secondary structure prediction server suggested that the prepro-region consists of three α-helices (Fig. 2B) (Cole et al. 2008; Chahal et al. 2017). However, experimental circular dichroism (CD) data analyzed by K2D3 prediction program revealed that only about 7% of the prepro-region consists of α-helices, while approximately 39% forms β-strands (Lin-Cereghino et al. 2013; Chahal et al. 2017). Based on these CD measurements, Chahal et al. proposed two potential structural models. Both are constituted of five β-strands and one α-helix situated between the fourth and the fifth β-strand (Fig. 2C). α-helix is suggested to be located either above the fourth and fifth, or rather above the first β-strand (Chahal et al. 2017) (Fig. 2C). By using the latest AlphaFold3 structure prediction (Abramson et al. 2024), we also found that the prepro-region is highly flexible, but with two anti-parallel β-strands between D49 and D67, followed by a C-terminal α-helix (Fig. 2D).

As discussed later in this review, Lin-Cereghino et al. analyzed mutated and truncated MFα prepro-regions with varying secretion efficiencies, suggesting that these differences might result from preferred or suboptimal tertiary structures (Lin-Cereghino et al. 2013). However, a comparison of CD data and secretion efficiencies of two reporter proteins revealed no clear correlation between specific tertiary structures and secretion outcomes (Chahal et al. 2017). The structure–function relationship likely involves a balance between highly flexible and more structured regions. Further investigation is needed to understand how these different intramolecular structures support protein secretion in vivo, particularly considering potential conformational changes in the unstructured regions when interacting with key factors of the protein secretion pathway.

### MFα signal sequences from different organisms

**Fig. 3:**
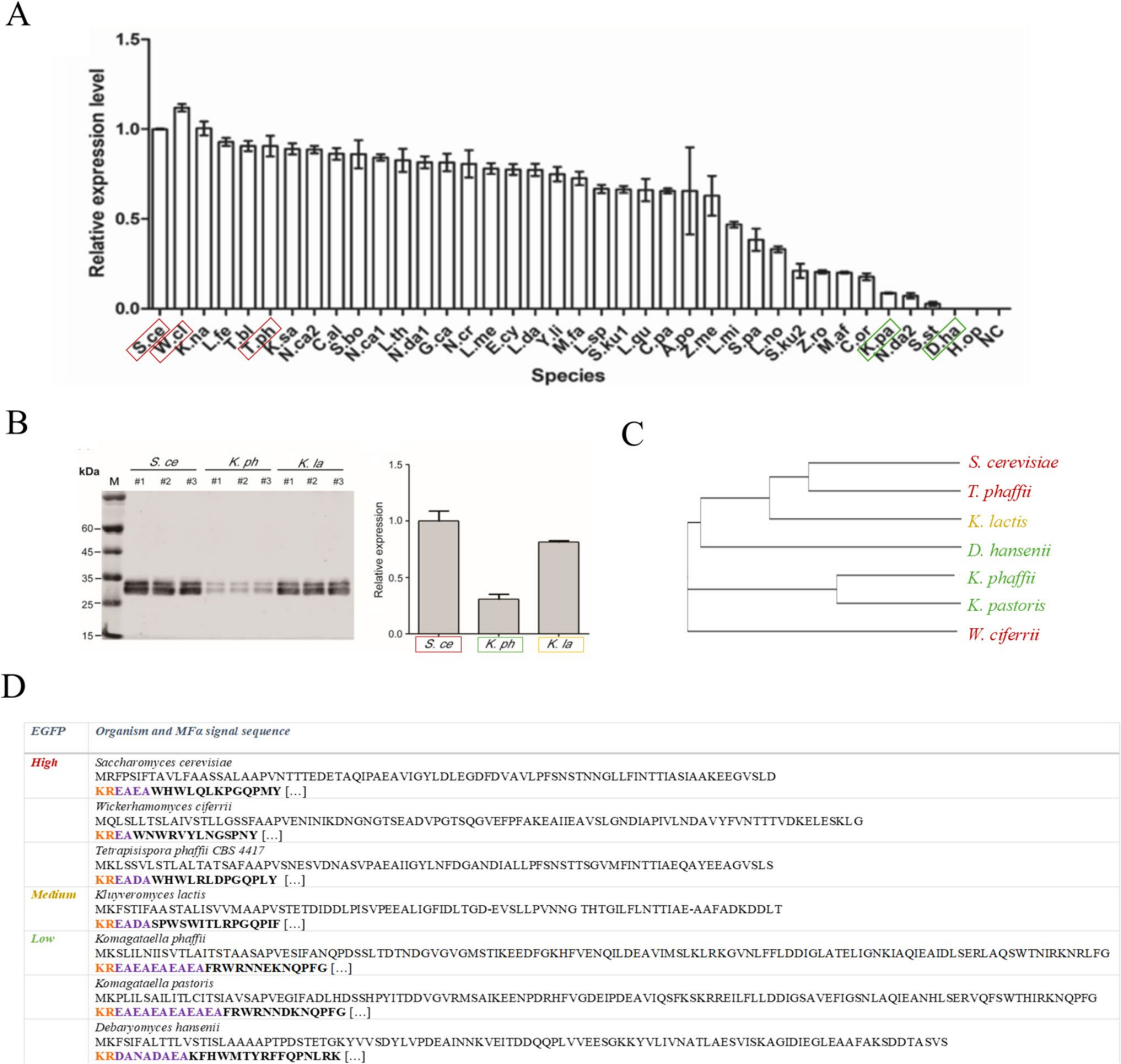
MFα secretion signal sequences from different yeasts*.* **A** Comparison of EGFP secretion and expression in *K. phaffii* mediated by α-MF secretion signals from different yeast species. Highlighted species include *S. cerevisiae* (S.ce), *W. ciferrii* (W.cl), *T. phaffii* CBS 4417 (T.ph), *K. pastoris* (K.pa), and *D. hansenii* CBS767 (D.ha). **B** Additional comparison between *S. cerevisiae* (S.ce), *K. phaffii* (K.ph), and *K. lactis* (K.la). Images were adapted from Zou et al. (2022). **C** Phylogenetic tree of the selected species constructed using ClustalW for sequence alignment and visualized with iTOL (Wigginton and Abecasis 2005). Branch lengths represent evolutionary distances, illustrating the relationships among the species analyzed. **D** Full-length secretion signal sequences of *S. cerevisiae*, *W. ciferrii*, *T. phaffii* CBS 4417, *K. lactis*, *K. phaffii*, *K. pastoris*, and *D. hansenii*. The sequences are categorized based on their ability to mediate high, medium, or low EGFP secretion in *K. phaffii*. Kex2 recognition sites are highlighted in orange, and DPAPase A endopeptidase recognition sites in purple

As in *S. cerevisiae*, mating in most yeast species is facilitated by the secretion of short peptide pheromones, which are produced and released by the cells to signal other cells in the mating process. While mating pheromones and their respective signal sequences have been identified in a limited number of fungi through genetic or biochemical studies, their short sequences and low conservation across species hinder the identification of homologous sequences in many fungi (Srikant et al. 2021). Despite these limitations, the NCBI database contains 39 additional α-MF genes from other yeast species, in addition to those of *S. cerevisiae* (Zou et al. 2022). Hypothesizing that endogenous signal sequences may enhance recombinant protein secretion, Zou et al. (2022) tested this hypothesis using Enhanced Green Fluorescent Protein (EGFP) as a model protein in *K. phaffii* (Zou et al. 2022). Their study evaluated 40 MFα signal sequences from different yeast species alongside 32 native *K. phaffii* signal peptides. The results showed that 38 of the 40 MFα signals, and 24 of the 32 native peptides successfully mediated protein secretion, though with varying efficiency. Notably, the MFα signal from *S. cerevisiae* demonstrated the highest secretion efficiency, followed by those from *Wickerhamomyces ciferrii*, and other non-model yeast (Zou et al. 2022). The data presented by Zou et al. (2022) highlight the conserved yet variable nature of MFα signals and show that species-specific variations severely impact secretion efficiency. Although additional data would be required for a comprehensive analysis, a comparative analysis of MFα signal sequences with high and low secretion efficiencies provides interesting insights into key sequence features influencing secretion dynamics in *K. phaffii*. We analyzed some MFα signal sequences associated with high or medium EGFP secretion (*S. cerevisiae*, *Wickerhamomyces ciferrii*, *Tetrapisispora phaffii*, and *Kluveromyces lactis*) and compared them to those yielding low EGFP secretion (*K. phaffii*, *Komagataella pastoris*, and *Debaryomyces hansenii*) (Zou et al. 2022) (Fig. 3C). The phylogenetic analysis of the signal sequences did not reveal a clear relationship between sequence similarity and secretion efficiency, as sequences associated with high and low secretion were not more closely related (Fig. 3C). Instead, differences in the recognition and cleavage sites for Kex2 and Ste13 proteases emerged as potential contributors to secretion efficiency (Fig. 3D). Notably, the Ste13 recognition sites, composed of Glu-Ala (EA) dipeptide repeats, displayed variation between the signal sequences. For the most efficient MFα variants from *S. cerevisiae*, *W. ciferrii*, *T. phaffii*, and *K. lactis*, a single or double EA dipeptide repeat was identified, respectively (Fig. 3D). In contrast, reduced secretion efficiency may correlate with the longer EA repeats present in the MFα signal sequences of *Komagataella* species. For the non-secreting variant from *D. hansenii*, a unique DANADAEA sequence replaces the EAEA or DAEA motifs typically found in *S. cerevisiae* (Julius et al. 1983; Voos and Stevens 1998) and other high-secretion variants (Zou et al. 2022). Additionally, the activity of the Kex2 endopeptidase is influenced not only by the core dibasic residues at the P2 and P1 positions (typically Lys-Arg, or KR) but also by the upstream residues, particularly at the P4 and P3 positions. It is well established that the presence of leucine at P4 is critical for Kex2 recognition, and motifs such as LXXR, LXRR, and LXKR are known to promote efficient cleavage and subsequent secretion in *S. cerevisiae* (Manfredi et al. 2016; Suzuki 2020; Kim et al. 2023). Variations in these upstream residues among the MFα signal sequences compared in this study likely contribute to the observed differences in secretion efficiency. For instance, Manfredi et al. (2016) demonstrated that substitution of the P3 aspartate with tyrosine improved Kex2 cleavage kinetics, and Kim et al. (2023) further confirmed that engineering the P3 and P4 positions significantly affects in vivo processing. These findings underscore the importance of the extended cleavage context, beyond just the KR motif, in modulating secretion efficiency in yeast expression systems.

### Insights into the molecular processing of the MFα signal peptide

Protease and peptidase recognition sequences generally consist of 1 to 4 amino acids, with residues upstream of the cleavage site designated as P4 to P1 and those downstream as P1′ to P4′. These sequences are highly specific, as proteases and peptidases exhibit strong preferences for particular amino acids surrounding the cleavage region. Even minor alterations can disrupt this specificity, often resulting in incorrect processing. Processing errors of signal peptides can have two major consequences: reduced protein secretion efficiency and retention of unwanted amino acids at the N-terminus of the mature protein.

In the case of *MFα* signal sequence processing, three proteases are involved: an as-yet unidentified ER protease, Kex2, and Ste13. Since Kex2 functions early in the secretory pathway in the Golgi, inefficient processing by this protease rarely results in N-terminally extended protein variants being secreted into the culture supernatant. Instead, Kex2 plays a pivotal role in secretion efficiency: deletion of the *KEX2* gene can lead to the accumulation of hyperglycosylated, unprocessed pro-proteins, which significantly impairs secretion of target proteins in *S. cerevisiae* (Fabre et al. 1991; Kjeldsen et al. 1996; Werten and de Wolf 2005; Hou et al. 2012). Interestingly, to some extent, the loss of Kex2 function can be compensated by Yps1, a protease from the yapsin family that is anchored to the plasma membrane via a GPI-anchor (glycosylphosphatidylinositol anchor). Yps1 has been shown to process secreted gelatin in *K. phaffii* even in the absence of *KEX2* (Werten and de Wolf, 2005). This suggests an alternative route for proteolytic processing under Kex2-deficient conditions.

Efforts to enhance protein secretion have explored modifying Kex2 localization. Actively re-localizing Kex2 from the Golgi to the ER has been shown to improve the secretion of recombinant human insulin-like growth factor-1 (IGF-1) in *S. cerevisiae* (ChaudhuriI et al. 1992). Several studies have also investigated the effects of amino acid substitutions within the Kex2 cleavage site. These substitutions can alter recombinant protein secretion by modifying protein structure or accessibility to Kex2 cleavage (Von Heijne 1984; Rockwell et al. 2002). Notably, the P1 site (R85) in the dibasic K84-R85 sequence tolerates minimal substitutions, with arginine (R85) being replaceable only by lysine (K85) (Von Heijne 1984; Rockwell et al. 2002). In contrast, the P2 site accommodates basic residues such as lysine and arginine equally well (Bevan et al. 1998). Subsequent studies have shown that the P1-P4 sites predominantly consist of apolar amino acid residues (Yang et al. 2013). This observation suggests that specific sequences are preferentially cleaved at these positions, potentially linked to the active site of Kex2 protease (Bader et al. 2008). Additionally, earlier evidence indicates that residues located downstream of the Kex2 cleavage site—especially in the negatively charged spacer region preceding the mature protein—are also significant (Zsebo et al. 1986; Kjeldsen et al. 1996). A study of the Kex2 protease structure (PDB: 1R64) revealed that the S4 to S1′ subsites play a critical role in substrate recognition and cleavage, corresponding to the P4 to P1′ positions of the substrate they bind (Manfredi et al. 2016). The selectivity of Kex2 is influenced by residues at the P3 position, with negatively charged residues reducing affinity, while aromatic residues enhance it. These in vitro experiments conducted with purified Kex2 and chemically synthesized peptide substrates suggest that further research is needed to understand how these substitutions affect protein secretion in whole cells.

In this context, the specificity of Kex2 protease was shown to be crucial for recombinant protein production and secretion. For example, site-saturation mutagenesis of the P1′ position in *K. phaffii* yielded varying results depending on the reporter protein. The fluorescent protein Venus showed highest secretion with S or A at P1′, while luciferase exhibited higher luminescence with N or K (Yang et al. 2013). Interestingly, multicopy integrations of a gene or DNA sequence in the genome played not only a role in enhancing yields, but also showed differences in secretion efficiency, which P1′ amino acid led to better secretion. For the defensin-derived peptide NZ2114, a Phe substitution at the P1′ site increased the yield from 2.39 to 4.81 g/L (Jin et al. 2023). Similarly, Rakestraw et al. (2009) substituting Glu with Ala or Val at the P1′ position increased G-CSF production, with further enhancement to 100 mg/L in a Mut^S^ host (Aggarwal and Mishra 2020). Even though these data are highly interesting, it remains unclear whether these modifications affect correct protein processing.

Because Ste13 functions in the late Golgi or post-Golgi compartments, specifically in the trans-Golgi network (TGN) or secretory vesicles, its inefficiency has a less dramatic impact on secretion efficiency compared to Kex2. Instead, Ste13 misprocessing primarily leads to the retention of EAEA overhangs at the N-terminus of the secreted protein, which can negatively affect protein function or activity (Rieder et al. 2021) and, in some cases, lead to protein aggregation (Nogueira et al. 2014). Such issues are particularly problematic for industrial and therapeutic applications, where they can complicate regulatory approval.

In *K. phaffii*, the endogenous Ste13 protease processes the MFα leader sequence less efficiently than its counterpart in *S. cerevisiae* (Puxbaum et al. 2015). A common strategy to prevent incorrect Ste13 processing is the removal of the EAEA sequence, as long as Kex2 cleavage at the KR sequence remains unaffected. This approach has been successfully applied to enable the secretion of several proteins in *K. phaffii*, including human plasminogen (Joshi and Sahni 2010)**,** insulin Aspart precursor (Kurniatin et al. 2019), *Pisum sativum* defensin 1 (rPsd1) (Cabral et al. 2003), human growth hormone (hGH) (Eurwilaichitr et al. 2002). Similarly, eliminating the Glu-Ala-Glu-Ala spacer peptide from the prepro signal sequence of MFα enabled the successful secretion of properly processed brazzein isoforms in *S. cerevisiae* (Poirier et al. 2012). However, direct comparisons have also shown that the removal of the EAEA sequence can negatively affect secretion efficiency, as demonstrated by a study on extracellular alpha-amylase in *S. cerevisiae*, where secretion was decreased (Southgate et al. 1993). As discussed earlier, the positions of the P1′ and P2′ residues are crucial for correct Kex2 processing. When the EAEA sequence is removed, the N-terminal amino acids of the target protein become the new P1′ and P2′ positions for Kex2 cleavage. This shift plays a critical role in whether or not the leader sequence is processed by Kex2. The importance of this has been highlighted by Zsebo et al. (1986), where they showed that the Glu-Ala-Glu-Ala region enhances Kex2 cleavage efficiency at the KR site within the MFα-prepro-α-interferon protein (Zsebo et al. 1986). Additionally, other studies have demonstrated that a proline at the P1′ position impedes Kex2 processing, as for example shown for human Interferon-lambda (Xie et al. 2007) or human granulocyte colony-stimulating factor (Aggarwal and Mishra 2020).

Despite its inefficiency, Ste13 appears to be less critical for secretion efficiency than Kex2, as proteins with minor N-terminal extensions resulting from incomplete Ste13 processing can still be successfully overproduced and secreted (Eurwilaichitr et al. 2002; Mateljak et al. 2017). Interestingly, extending the N-terminus with short functional peptides or retaining the native EAEA residues has been shown to enhance protein titres, as demonstrated for vaccine antigens, where titres increased by up to 76% (Dalvie et al. 2022). This strategy has also proven useful for producing other proteins, such as insulin, through codon optimization and the removal of unnecessary spacer sequences.

## Optimization of protein secretion employing the MFα signal sequence

Even though the MFα signal sequence is the most often used signal sequence in protein secretion, several challenges can limit the efficient export of recombinant proteins. One significant bottleneck arises from the pro-region of MFα, which is prone to aggregation within the endoplasmic reticulum (ER), as shown by Barrero et al. (2018). This aggregation can impair proper protein folding and disrupt secretion. In some cases, the full-length MFα prepro signal sequence prevents the secretion of recombinant proteins, while secretion is successful when only the pre-MFα signal is used (Zhao et al. 2009). This indicates that the pro-region, although beneficial for certain proteins, can also interfere with folding or the secretory pathway. Furthermore, proteins fused to the full MFα-prepro signal sequence are sometimes retained in the ER or Golgi apparatus, blocking their transport to the extracellular space (Porro et al. 2005). To address these limitations, strategies have been developed, including modifications to the MFα-prepro signal sequence and cell engineering approaches, as discussed in the following chapter.

### Mutational analysis of the pre and pro leader signal sequence

When studying the impact of mutations on secretion, selecting an appropriate reporter protein is a critical first step. Secretion bottlenecks are often highly specific to the target protein, making the selection of a suitable model protein crucial. It is essential to choose a well-characterized protein that is known to encounter secretion challenges, as this will provide valuable insights into the underlying mechanisms. Several reporter proteins have emerged as effective models for studying secretion, including GFP and EGFP (Roh et al. 2013), β-galactosidase (Tan et al. 2002), horseradish peroxidase (HRP) (Krainer et al. 2016), laccase (Antošová and Sychrová 2016), and *Candida antarctica* lipase B (CalB) (Eom et al. 2013; Vadhana et al. 2013; Xiao et al. 2023). These proteins are widely used in secretion studies due to their high detectability with standard, robust assays, making them invaluable tools for investigating protein trafficking, folding, and export.

**Fig. 4:**
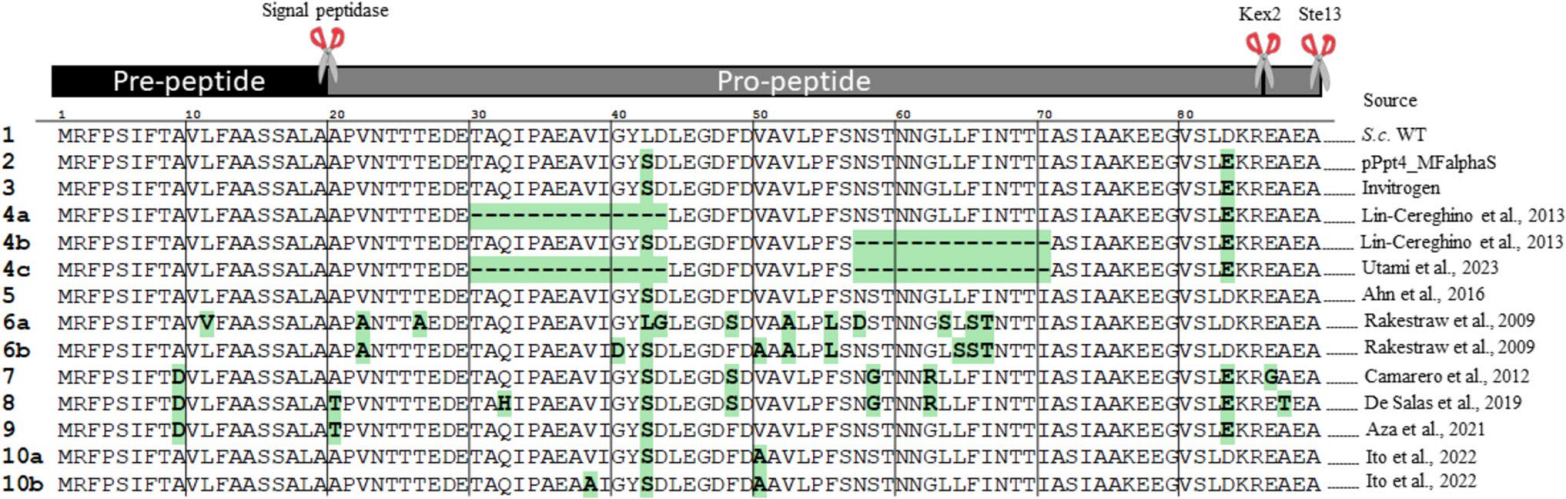
Overview of the key mutations and variants of the MFα signal sequence that enhance protein secretion (figure adapted from Merkaš et al. (2025)). The corresponding DNA sequences for these variants are available in Merkaš et al. (2025)

When broadly trying to elucidate the function and stability of a protein or peptide, mutational analysis is an invaluable tool (Gundry and Vijg 2012). This technique has been effectively applied to the MFα signal sequence in yeasts, where iterative mutagenesis and selection have been used to optimize secretion efficiency (Merkaš et al. 2025). By introducing targeted mutations in specific amino acid residues, researchers have achieved notable improvements in secretion rates, protein yield, and stability, positioning MFα as a versatile secretion leader for diverse biotechnological applications (Fig. 4). For instance, Camarero et al. (2012) reported that mutations such as A9D, F48S, and S58G significantly enhanced laccase secretion in *S. cerevisiae*. Among these, A9D boosted secretion up to 13-fold, while F48S improved secretion by altering critical hydrophobic interactions during ER processing (Camarero et al. 2012). Similary Rakestraw et al. (2009) identified mutations like V22 A, L42S, and V50 A, which enhanced the secretion of single-chain variable fragment (scFv) antibodies in *S. cerevisiae*. Remarkably, Val50 Ala, initially identified in *S. cerevisiae*, was later shown to enhance antibody secretion in *K. phaffii*, highlighting the cross-species relevance of these mutations (Ito et al. 2022).

Beyond individual mutations, structural modifications of the MFα pro-region have played a pivotal role in optimizing secretion. Deleting the third alpha helix (amino acids 57–70) improved the secretion of HRP and CalB by over 50% (Lin-Cereghino et al. 2013), while further deletions of residues 30–43 and 57–70 increased HRP expression by 137% and enabled high-quality production of CRM197, a key vaccine component (Aw et al. 2021). Combining structural deletions with codon-optimized target genes has also significantly enhanced secretion in therapeutic applications, such as granulocyte colony-stimulating factor using a Δ57–70 deletion in the MFα pro-region for improved secretion of granulocyte colony-stimulating factor (Aggarwal and Mishra 2020) and truncated MFα variants for improved secretion of human insulin precursors in *K. phaffii* (Utami et al. 2023).

Codon optimization is a highly effective strategy for enhancing secretion by improving translational efficiency through alignment of codon usage with the host organism’s tRNA availability. Combining codon optimization with a synthetic MFα leader sequence enriched with glutamic acid and alanine has been shown to increase bacterial phytase secretion by over five-fold (Xiong et al. 2005). A similar approach applied to CalB boosted its secretion from 3 to 12 U/mL (Ahn et al. 2016). The hydrophobicity and polarity of the MFα pro-region also play critical roles in secretion efficiency, with mutations that stabilize the N- and C-termini of the pro-region enhancing secretion, while those in the interior further improve performance if structural stability is maintained (Chahal et al. 2017).

The concept of mutation synergy has been central to many of these advancements. A combination of mutations—such as D9 A, A20 T, L42S, and D83E—has been shown to enhance the secretion of fungal oxidoreductases and hydrolases (Aza et al. 2021). Directed evolution experiments further validated the importance of synergistic mutations. The MFα signal variants αppS8 and αppS4, which combined mutations including V22 A, G40D, L42S, and V50 A, significantly improved secretion efficiency of single-chain variable fragment (scFv) antibodies in *S. cerevisiae*. Importantly, these mutations enhanced protein trafficking without negatively affecting mRNA levels or growth rates (Rakestraw et al. 2009; Ito et al. 2022). However, excessive mutation complexity sometimes resulted in diminishing efficiency, underscoring the need for a balanced approach to engineering (Teufl et al. 2022).

Recent advances in high-throughput screening and machine learning have expanded the scope of MFα optimization. De Salas et al. (2019) screened over 4000 MFα variants and identified mutations such as A20 T and Q32H that increased laccase secretion by 8.5-fold in *K. phaffii*. These findings underscore the power of combining automated design and directed evolution to unlock new levels of secretion efficiency in yeast expression systems.

### Combining the MFα with other signal sequences

In addition to the strategies discussed above, combining the MFα signal sequence with other signal sequences offers another promising approach to enhance secretion efficiency in yeast. These combinatorial strategies aim to create chimeric or hybrid leader sequences that leverage the strengths of both components, improving secretion efficiency, host organism compatibility, and recombinant protein yields. One such strategy involves the pre-Ost1 signal leader from *S. cerevisiae*, which directs co-translational translocation (Willer et al. 2008; Forte et al. 2011). Fitzgerald and Glick (2014) investigated the secretion of monomeric superfolder GFP (msGFP) in *S. cerevisiae* and identified two key obstacles: inefficient endoplasmic reticulum (ER) translocation, and vacuolar targeting via Vps10, a vacuolar sorting protein (Fitzgerald and Glick 2014). Vps10, a complex protein with two functional domains, plays a pivotal role in cellular sorting by targeting foreign proteins, such as msGFP, for degradation in the vacuole (Jørgensen et al. 1999). Truncating Vps10 to remove domain 1, which is responsible for this vacuolar targeting, enhanced msGFP secretion without affecting the sorting of essential vacuolar hydrolases (Fitzgerald and Glick 2014). Additionally, a hybrid signal sequence combining the pre-*Ost1* leader with the pro-MFα region was established, which significantly improving ER export and secretion of msGFP.

Building on this work, subsequent studies applied the hybrid pre-Ost1 pro-MFα signal sequence to secrete other proteins in *S. cerevisiae*. For example, the secretion of the tetrameric far-red fluorescent protein E2-Crimson and the enzyme lipase 2 from *Bacillus thermocatenulatus* (BTL2) showed a 20-fold and tenfold increase in efficiency, respectively, compared to the traditional prepro MFα sequence (Barrero et al. 2018). This hybrid leader also enhanced extracellular CelA activity by 2.7- (Besada-Lombana and Da Silva 2019). Further optimization, such as substituting leucine with serine at position 42 in the pro-MFα region, reduced protein aggregation in the ER lumen and further boosted secretion (Barrero et al. 2018). Encouraged by these results, researchers scaled up the application of the hybrid signal sequence using bioreactors, an essential step for industrial applications. In shake-flask experiments, the secretion efficiencies of E2-Crimson, BTL2, and lipase from *Rhizopus oryzae* (ROL) translated well to bioreactor conditions, demonstrating the potential of the pre-Ost1 leader as a viable alternative to MFα for industrial-scale protein production (Barrero et al. 2021).

However, not all proteins benefit from this hybrid approach. When the hybrid leader was used to express lytic polysaccharide monooxygenases (LPMOs)—enzymes essential for breaking down complex carbohydrates—incorrect N-terminal processing rendered the enzymes inactive. In contrast, the pre-Ost1 leader proved more effective for producing LPMOs in *K. phaffii*, resulting in properly processed, functional enzymes (Rieder et al. 2021). This outcome underscores a crucial point: while hybrid leaders can enhance secretion for many proteins, they are not universally applicable. The structural and functional requirements of individual proteins must be carefully considered when designing secretion strategies.

### Optimization by cell engineering and expression strategies

In addition to sequence-specific factors, several other elements influence the efficiency of recombinant protein secretion using MFα signal sequences, including the choice of expression strains, promoters, and cultivation conditions. Selecting an appropriate yeast strain is a first essential step, as secretion capabilities vary among species. *Saccharomyces cerevisiae*, the first yeast system employed for recombinant protein production in the 1980 s (Gellerforss et al. 1989), remains a preferred model organism due to its well-characterized genetics, ease of manipulation, and ability to perform post-translational modifications similar to those in higher eukaryotes. Other yeast systems have since been utilized for protein secretion, including *Schizosaccharomyces pombe* (Klein et al. 2014), *Candida boidinii* (Sakai et al. 1996), *Pichia methanolica* (Wang et al. 2004), *Hansenula polymorpha* (Gellissen et al. 1991), *Zygosaccharomyces bailii* (Branduardi et al. 2004), *Klyveromyces lactis* (Spohner et al. 2016), filamentous fungi *Aspergillus oryzae* (Camarero et al. 2012), and the dimorphic species *Arxula adeninivorans* (Wartmann et al. 2002), *Yarrowia lipolytica* (Madzak et al. 2004), and most importantly *K. phaffii* (Cereghino & Cregg 2000; Liu et al. 2022; Barone et al. 2023). Among these, *K. phaffii* is notable for its high-density growth and robust secretion capabilities, especially when using strains with *HOC1* open-reading-frame truncations (Claes et al. 2024), making it ideal for recombinant protein production using MFα signal sequences. Promoter selection also plays a crucial role in secretion efficiency. Strong, constitutive promoters such as *TEF* (translation elongation factor), *PGK* (phosphoglycerate kinase), *ADH1* (alcohol dehydrogenase 1), and *GAP* (glyceraldehyde-3-phosphate dehydrogenase) are commonly used, particularly in *S. cerevisiae* (Gomes et al. 2018). In *K. phaffii*, methanol-inducible promoters like *AOX1*, *CAT1*, and *HpFMD* support high protein expression and secretion (Vassileva et al. 2001; Zhang et al. 2009; Roongsawang et al. 2016; Vogl et al. (2016, 2020)). Notably, while *GAP* can drive constitutive expression under methanol-free conditions such as glycerol-based media, *CAT1* and *HpFMD* are regulated promoters that can achieve high expression levels under derepressed conditions (e.g., when glucose is depleted), enabling tight control of induction without methanol. This makes them attractive alternatives in industrial settings seeking to avoid methanol while maintaining regulatory flexibility.

Optimizing the yeast secretion pathway can improve efficiency, but this process is complex and involves balancing multiple factors such as secretion efficiency, glycosylation, cellular stress, and competition for secretion machinery. High levels of protein expression can induce stress, which negatively affects growth and viability, while competition for secretion resources may reduce overall efficiency (Mattanovich et al. 2012). An intriguing approach to encounter the complexity and the limited energy resources of the yeast metabolism has been presented by Li et al. The developed genome-wide model pcSecYeast allows the identification of over-expression targets including metabolic genes as well as protein synthesis and secretion related genes, resulting in an enhanced protein secretion (Li et al. 2022).

As already mentioned, improper folding of the pro-region in the MFα signal can lead to aggregation in the ER and prevent protein secretion (Barrero et al. 2018). Some recombinant proteins fail to be secreted when fused to the full-length MFα-pro-pro signal but are successfully secreted with only the pre-MFα signal (Zhao et al. 2009), while others are retained in the ER or Golgi, preventing extracellular export (Porro et al. 2005). Such accumulation in the ER triggers the unfolded protein response (UPR), a crucial cellular mechanism that addresses the buildup of misfolded proteins in the ER. Initially recognized as a stress response, the UPR not only enhances the production of ER-resident chaperones to aid protein folding but also plays a broader role in maintaining ER and cellular homeostasis. It coordinates key processes across the secretory pathway, including lipid metabolism, protein translocation, ER-associated degradation (ERAD), and transport between the ER and Golgi apparatus (Radanović and Ernst 2021). Engineering UPR mechanisms can significantly enhance the secretion of recombinant proteins by alleviating ER stress and improving protein folding and processing. Strategies for this include the overexpression of ER chaperones (Prabhu et al. 2018), optimization of translocation pathways (Zhu et al. 2023), and co-expression with cytosolic heat shock proteins (HSPs), which assist in folding proteins prior to ER entry (Zhu et al. 2023). For a comprehensive review of these approaches, we also refer the reader to Raschmanová et al. (2021).

As previously discussed, missorting of the signal peptide significantly impacts protein secretion. For instance, post-translational translocation, as seen with the MFα signal sequence, can lead to premature protein folding, as observed for msGFP (Fitzgerald and Glick 2014). This premature folding causes recombinant proteins to accumulate in the cytoplasm, preventing their proper translocation to the endoplasmic reticulum (ER) and potentially clogging the translocon pores (Ast et al. 2016). A successful strain engineering strategy to address this issue involved truncation of VPS10, which is responsible for binding and missorting heterologous proteins into the vacuole (Fitzgerald and Glick 2014). RNA-seq analysis of *K. phaffii* cells overproducing and secreting EGFP revealed significant upregulation of several subunits of the Sec complex, including Sec61p, Sbh1p, Sss1p, Sec66p, and Sec72p (Wang et al. 2024). Active overexpression of the corresponding genes resulted in a 16%, 58%, 16%, and 17% increase in α-amylase activity in strains overexpressing Sss1, Sbh1, Sec66, and Sec72, respectively. Post-translational glycosylation in the ER further adds complexity to the secretion process. The pro-region of the MFα signal contains three N-linked glycosylation sites critical for efficient transport through the secretory pathway. It aids in proper folding, facilitates ER-to-Golgi transport, and ensures efficient processing of the precursor protein. These findings are particularly relevant when designing expression systems for heterologous proteins in yeast (Han and Yu 2015). Mutations in these sites can significantly reduce secretion, as demonstrated in *S. cerevisiae* by decreased insulin precursor secretion (Kjeldsen et al. (1996, 1999)) and in *K. phaffii* by impaired eGFP secretion (Dai et al. 2024), although secretion was not entirely abolished. In addition, in *S. cerevisiae*, deletions of the genes encoding Vps5 and Vps17, involved in Golgi-endosome trafficking, significantly enhanced secretion and reduced intracellular retention of the target protein fungal α-amylase, offering promising engineering strategies for improved secretion efficiency (Huang et al. 2018).

A recent study in *S. cerevisiae* highlighted the concept of “induction sweet spots” for hard-to-secrete proteins, where optimal induction levels vary based on specific proteins and growth conditions. Exceeding these sweet spots can result in “secretion burnout,” characterized by high cellular stress elevated levels of UPR, slower growth, and reduced secretion efficiency (Sosa-Carrillo et al. 2023). Using engineered yeast strains, light-responsive promoters, and an automated turbidostat platform, researchers achieved a 70% increase in secreted protein levels, underscoring the importance of optimizing overall secretion mechanisms rather than focusing solely on the leader sequence. Taken together, efficient secretion of recombinant proteins using MFα signal sequences requires a multifaceted approach, balancing sequence-specific features with strain selection, promoter optimization, and control of stress responses, particularly through enhanced glycosylation and unfolded protein response mechanisms.

Recent studies have explored the use of machine learning and high-throughput screening to optimize signal peptides in *Saccharomyces cerevisiae*. For example, tools like the Signal Peptide Optimization Tool (SPOT) and engineered α-factor leader sequences have been shown to significantly enhance heterologous protein secretion (Mori et al. 2015).

## Conclusion and outlook

The MFα signal sequence has long been central to yeast-based protein secretion, demonstrating remarkable versatility across diverse applications, including enzyme production and therapeutic protein synthesis, and functionality in various yeast species. Its robustness and effectiveness seem almost fortuitous, as no other signal sequence identified to date matches its efficiency, versatility, and consistency. Over the years, significant improvements have been made through modifications to the MFα sequence itself and advancements in strain engineering, which have enhanced secretion efficiency and expanded its potential applications. Notable solutions have included the fusion of different pre- and pro-leader sequences, exemplified by the pre-Ost1 and pro-MFα signal variants. Despite these advancements, secretion efficiency remains highly variable, even for proteins with similar structures, often necessitating tailored strategies for each protein (Obst et al. 2017). Furthermore, challenges such as susceptibility to missorting, incorrect processing, and competition for secretion machinery underscore the need for continued development of improved alternatives.

Estimating the success rate whether or not a recombinant protein can be secreted using the MFα or other signal sequences is challenging, as failures are rarely reported in the literature. Based on our own experience testing a wide range of recombinant proteins in the Glieder and Emmerstorfer-Augustin labs, we estimate the success rate to be around 30–60%. This suggests that a significant proportion of recombinant proteins either cannot be produced by the cell or fail to efficiently navigate the secretory pathway. To address these challenges and identify alternative universal secretion leader sequences, novel signal sequences are currently under investigation. Notably, the Pir1 and PAS_chr3_0030 signal sequences have shown promising results (Karaoglan et al. 2014). The Pir1 sequence has demonstrated superior performance in extracellular enzyme production, while PAS_chr3_0030 has facilitated efficient secretion of various industrially relevant enzymes in *K. phaffii* (Shen et al. 2022). A major focus of ongoing research is the identification of species-specific or engineered hybrid signal peptides that can overcome the secretion challenges unique to specific host systems. A key direction in ongoing research is the development of species-specific or engineered hybrid signal peptides tailored to the host system. For example, O’Riordan et al. (2024) introduced the Yeast Modular Cloning (MoClo) Toolkit, which allows systematic testing of different combinations of pre- and pro-signal sequences to enhance secretion. These screening approaches are particularly valuable, as secretion efficiency often depends on the specific protein being expressed. Additionally, engineered bacterial signal sequences are being optimized to better accommodate the diverse demands of heterologous protein expression in prokaryotic systems, often enhancing protein folding and export efficiency through the Sec pathway (Freudl 2018). Similarly, in mammalian cells, secretion processes rely heavily on SRP-dependent post-translational translocation (Lakkaraju et al. 2012). These targeted modifications aim to improve secretion efficiency and address bottlenecks in protein production for various host organisms.

Advances in bioinformatics and machine learning are accelerating the design and evaluation of synthetic signal peptides. Xue et al. (2023) conducted a comprehensive bioinformatics analysis of signal peptides in *S. cerevisiae* and identified conserved features associated with efficient protein secretion. Tools such as BastionHub, which analyzes substrates secreted by Gram-negative bacteria (Wang et al. 2021), EffHunter, which predicts effective protein candidates in fungal systems (Carreón-Anguiano et al. 2020), and TISIGNER.com (Bhandari et al. 2021) are facilitating these developments. Researchers are leveraging these platforms alongside high-throughput screening methods to identify thousands of potential signal sequences. These approaches enable the customization of sequences tailored to specific proteins or production conditions, with the ultimate goal of predictably enhancing secretion efficiency across diverse host organisms and applications.

Also, the exploration of non-classical secretion pathways is emerging as a promising approach to overcome bottlenecks in traditional protein production systems. Unlike conventional signal sequences that direct proteins through the endoplasmic reticulum and Golgi apparatus, these novel pathways bypass these organelles, enhancing yields of complex proteins that are otherwise challenging to express (Kang and Zhang 2020). Complementary strategies such as codon optimization, co-expression of molecular chaperones, and the use of protease-deficient strains can further improve secretion efficiency. Together, these innovations represent the next generation of signal sequence research, enabling researchers to push the boundaries of protein expression systems. By leveraging novel signal peptides, optimizing secretion pathways, and employing synthetic biology tools, scientists are paving the way for more efficient biotechnological applications, from industrial enzyme production to biopharmaceuticals.

While this review highlights the most effective MFα sequence variants, the unpredictability of secretion efficiency remains a major challenge in identifying a truly universal secretion leader sequence. Overcoming this hurdle will require more systematic studies and the development of high-throughput approaches to discover and optimize novel signal sequences that can achieve or surpass the performance of current variants. In summary, the integration of innovative signal sequences, advanced computational tools, and cutting-edge engineering strategies is transforming the field of protein secretion research. These efforts are bringing us closer to realizing highly efficient and customizable secretion systems, with the potential for significant breakthroughs in industrial enzyme production, biopharmaceuticals, and other applications. Further research will undoubtedly yield new insights, possibly leading to the discovery of a truly universal secretion leader sequence.

## Data Availability

No datasets were generated or analysed during the current study.

## References

[CR1] Abramson J, Adler J, Dunger J, Evans R, Green T, Pritzel A, Ronneberger O, Willmore L, Ballard AJ, Bambrick J, Bodenstein SW, Evans DA, Hung CC, O’Neill M, Reiman D, Tunyasuvunakool K, Wu Z, Žemgulytė A, Arvaniti E, Beattie C, Bertolli O, Bridgland A, Cherepanov A, Congreve M, Cowen-Rivers AI, Cowie A, Figurnov M, Fuchs FB, Gladman H, Jain R, Khan YA, Low CMR, Perlin K, Potapenko A, Savy P, Singh S, Stecula A, Thillaisundaram A, Tong C, Yakneen S, Zhong ED, Zielinski M, Žídek A, Bapst V, Kohli P, Jaderberg M, Hassabis D, Jumper JM (2024) Accurate structure prediction of biomolecular interactions with AlphaFold 3. Nature 630:493–500. 10.1038/s41586-024-07487-w 38718835 10.1038/s41586-024-07487-w PMC11168924

[CR2] Aggarwal S, Mishra S (2020) Differential role of segments of α-mating factor secretion signal in *Pichia**pastoris* towards granulocyte colony-stimulating factor emerging from a wild type or codon optimized copy of the gene. Microb Cell Fact 19:199. 10.1186/s12934-020-01460-8 33121493 10.1186/s12934-020-01460-8 PMC7597063

[CR3] Ahn J, Jang MJ, Ang KS, Lee H, Choi ES, Lee DY (2016) Codon optimization of *Saccharomyces**cerevisiae* mating factor alpha prepro-leader to improve recombinant protein production in *Pichia**pastoris*. Biotechnol Lett 38:2137–2143. 10.1007/s10529-016-2203-3 27613154 10.1007/s10529-016-2203-3

[CR4] Akeboshi H, Chiba Y, Kasahara Y, Takashiba M, Takaoka Y, Ohsawa M, Tajima Y, Kawashima I, Tsuji D, Itoh K, Sakuraba H, Jigami Y (2007) Production of recombinant β-hexosaminidase A, a potential enzyme for replacement therapy for Tay-Sachs and Sandhoff diseases, in the methylotrophic yeast *Ogataea**minuta*. Appl Environ Microbiol 73:4805–4812. 10.1128/AEM.00463-07 17557860 10.1128/AEM.00463-07 PMC1951009

[CR5] Antošová Z, Sychrová H (2016) Yeast hosts for the production of recombinant laccases: a review. Mol Biotechnol 58:93–116. 10.1007/s12033-015-9910-1 10.1007/s12033-015-9910-1 26698313

[CR6] Ast T, Michaelis S, Schuldiner M (2016) The protease Ste24 clears clogged translocons. Cell 164:103–114. 10.1016/j.cell.2015.11.053 26771486 10.1016/j.cell.2015.11.053 PMC4715265

[CR7] Aw R, Ashik MR, Islam AAZM, Khan I, Mainuddin M, Islam MA, Ahasan MM, Polizzi KM (2021) Production and purification of an active CRM197 in *Pichia**pastoris* and its immunological characterization using a Vi-typhoid antigen vaccine. Vaccine 39:7379–7386. 10.1016/j.vaccine.2021.10.083 34774362 10.1016/j.vaccine.2021.10.083

[CR8] Aza P, Molpeceres G, de Salas F, Camarero S (2021) Design of an improved universal signal peptide based on the α-factor mating secretion signal for enzyme production in yeast. Cell Mol Life Sci 78:3691–3707. 10.1007/s00018-021-03793-y 33687500 10.1007/s00018-021-03793-y PMC8038962

[CR9] Bader O, Krauke Y, Hube B (2008) Processing of predicted substrates of fungal Kex2 proteinases from Candida albicans, C. glabrata, Saccharomyces cerevisiae and Pichia pastoris. BMC Microbiol 8:116. 10.1186/1471-2180-8-116 18625069 10.1186/1471-2180-8-116 PMC2515848

[CR10] Baker D, Shiau AK, Agard DA (1993) The role of pro regions in protein folding. Curr Opin Cell Biol 5:966–970. 10.1016/0955-0674(93)90078-5 8129949 10.1016/0955-0674(93)90078-5

[CR11] Barbieri G, Simon J, Lupusella CR, Pereira F, Elia F, Meyer H, Schuldiner M, Hanes SD, Nguyen D, Helms V, Römisch K (2023) Sec61 channel subunit Sbh1/Sec61β promotes ER translocation of proteins with suboptimal targeting sequences and is fine-tuned by phosphorylation. J Biol Chem 299:102895. 10.1016/j.jbc.2023.102895 36639027 10.1016/j.jbc.2023.102895 PMC9947333

[CR12] Bar-Nun S, Kreibich G, Adesnik M, Alterman L, Negishi M, Sabatini DD (1980) Synthesis and insertion of cytochrome P-450 into endoplasmic reticulum membranes. Proc Natl Acad Sci U S A 77:965–969. 10.1073/pnas.77.2.96 6767247 10.1073/pnas.77.2.965 PMC348404

[CR13] Barone GD, Emmerstorfer-Augustin A, Biundo A, Pisano I, Coccetti P, Mapelli V, Camattari A (2023) Industrial production of proteins with *Pichia**pastoris*—*Komagataella**phaffii*. Biomolecules 13:441. 10.3390/biom13030441 36979376 10.3390/biom13030441 PMC10046876

[CR14] Barrero JJ, Casler JC, Valero F, Ferrer P, Glick BS (2018) An improved secretion signal enhances the secretion of model proteins from *Pichia**pastoris*. Microb Cell Fact 17:161. 10.1186/s12934-018-1009 30314480 10.1186/s12934-018-1009-5 PMC6182871

[CR15] Barrero JJ, Pagazartaundua A, Glick BS, Valero F, Ferrer P (2021) Bioreactor-scale cell performance and protein production can be substantially increased by using a secretion signal that drives co-translational translocation in *Pichia**pastoris*. N Biotechnol 60:85–95. 10.1016/j.nbt.2020.09.001 33045421 10.1016/j.nbt.2020.09.001 PMC7680431

[CR16] Besada-Lombana PB, Da Silva NA (2019) Engineering the early secretory pathway for increased protein secretion in *Saccharomyces**cerevisiae*. Metab Eng 55:142–151. 10.1016/j.ymben.2019.06.010 31220665 10.1016/j.ymben.2019.06.010

[CR17] Bevan A, Brenner C, Fuller RS (1998) Quantitative assessment of enzyme specificity *in vivo*: P 2 recognition by Kex2 protease defined in a genetic system. Proc Natl Acad Sci USA 95:10384–10389. 10.1073/pnas.95.18.10384 9724712 10.1073/pnas.95.18.10384 PMC27903

[CR18] Bhandari BK, Lim CS, Gardner PP (2021) TISIGNER.com: web services for improving recombinant protein production. Nucleic Acids Res 49:654–661. 10.1093/nar/gkab175 10.1093/nar/gkab175 PMC8265118 33744969

[CR19] Brake AJ, Merryweather JP, Coit DG, Heberlein UA, Masiarz FR, Mullenbach GT, Urdea MS, Valenzuela P, Barr PJ (1984) Alpha-factor-directed synthesis and secretion of mature foreign proteins in *Saccharomyces**cerevisiae*. Proc Natl Acad Sci USA 81:4642–4646. 10.1073/pnas.81.15.4642 6087338 10.1073/pnas.81.15.4642 PMC391546

[CR20] Branduardi P, Valli M, Brambilla L, Sauer M, Alberghina L, Porro D (2004) The yeast *Zygosaccharomyces**bailii*: a new host for heterologous protein production, secretion and for metabolic engineering applications. FEMS Yeast Res 4:493–504. 10.1016/S1567-1356(03)00200-9 14734030 10.1016/S1567-1356(03)00200-9

[CR21] Cabral KMS, Almeida MS, Valente AP, Almeida FCL, Kurtenbach E (2003) Production of the active antifungal Pisum sativum defensin 1 (Psd1) in *Pichia**pastoris*: overcoming the inefficiency of the STE13 protease. Protein Expr Purif 31:115–122. 10.1016/S1046-5928(03)00136-0 12963348 10.1016/s1046-5928(03)00136-0

[CR22] Camarero S, Pardo I, Cañas AI, Molina P, Record E, Martínez AT, Martínez MJ, Alcalde M (2012) Engineering platforms for directed evolution of laccase from *Pycnoporus**cinnabarinus*. Appl Environ Microbiol 78:1370–1384. 10.1128/AEM.07530-11 22210206 10.1128/AEM.07530-11 PMC3294479

[CR23] Carreón-Anguiano KG, Islas-Flores I, Vega-Arreguín J, Sáenz-Carbonell L, Canto-Canché B (2020) Effhunter: a tool for prediction of effector protein candidates in fungal proteomic databases. Biomolecules 10:712. 10.3390/biom10050712 32375409 10.3390/biom10050712 PMC7277995

[CR24] Cereghino JL, Cregg JM (2000) Heterologous protein expression in the methylotrophic yeast *Pichia**pastoris*. FEMS Microbiol Rev 24:45–66. 10.1111/j.1574-6976.2000.tb00532.x 10640598 10.1111/j.1574-6976.2000.tb00532.x

[CR25] Chahal S, Wei P, Moua P, Park SPJ, Kwon J, Patel A, Vu AT, Catolico JA, Tsai YFT, Shaheen N, Chu TT, Tam V, Khan ZE, Joo HH, Lin-Cereghino J, Tsai JW, Lin-Cereghino GP (2017) Structural characterization of the α-mating factor prepro-peptide for secretion of recombinant proteins in *Pichia**pastoris*. Gene 598:50–62. 10.1016/j.gene.2016.10.040 27984193 10.1016/j.gene.2016.10.040

[CR26] ChaudhuriI B, Steube K, Stephan C (1992) The pro-region of the yeast prepro-α-factor is essential for membrane translocation of human insulin-like growth factor 1 *in**vivo*. Eur J Biochem 206:793–800. 10.1111/j.1432-1033.1992.tb16986.x 1606961 10.1111/j.1432-1033.1992.tb16986.x

[CR27] Claes K, Van Herpe D, Vanluchene R, Roels C, Van Moer B, Wyseure E, Vandewalle K et al (2024) OPENPichia: licence-free *Komagataella**phaffii* chassis strains and toolkit for protein expression. Nat Microbiol 9:864–876. 10.1038/s41564-023-01574-w 38443579 10.1038/s41564-023-01574-w PMC10914597

[CR28] Clare JJ, Romanos MA, Rayment FB, Rowedder JE, Smith MA, Payne MM, Sreekrishnab K, Henwooda CA (1991) Production of mouse epidermal growth factor in yeast: high-level secretion using *Pichia**pastoris* strains containing multiple gene copies. Gene 105:205–212. 10.1016/0378-1119(91)90152-2 1937016 10.1016/0378-1119(91)90152-2

[CR29] Cohen MJ, Chirico WJ, Lipke PN (2020) Through the back door: unconventional protein secretion. Cell Surf 6:100045. 10.1016/j.tcsw.2020.100045 33225116 10.1016/j.tcsw.2020.100045 PMC7666356

[CR30] Cole C, Barber JD, Barton GJ (2008) The Jpred 3 secondary structure prediction server. Nucleic Acids Res 36:197–201. 10.1093/nar/gkn238 10.1093/nar/gkn238 PMC2447793 18463136

[CR31] Dai H, Zhang C, Wu J, Tang Q, Xie Y, Yu Y, Lin Y, Huang Y (2024) Optimizing *Pichia**pastoris* protein secretion: role of N-linked glycosylation on the α-mating factor secretion signal leader. J Biotechnol 391:1–10. 10.1016/j.jbiotec.2024.04.008 38636846 10.1016/j.jbiotec.2024.04.008

[CR32] Dalvie NC, Naranjo CA, Rodriguez-Aponte SA, Johnston RS, Christopher Love J (2022) Steric accessibility of the N-terminus improves the titer and quality of recombinant proteins secreted from *Komagataella phaffii*. Microb Cell Fact 21:180. 10.1186/s12934-022-01905-2 36064410 10.1186/s12934-022-01905-2 PMC9444097

[CR33] De Salas F, Aza P, Gilabert JF, Santiago G, Kilic S, Sener ME, Vind J, Guallar V, Martínez AT, Camarero S (2019) Engineering of a fungal laccase to develop a robust, versatile and highly-expressed biocatalyst for sustainable chemistry. Green Chem 21:5374–5385. 10.1039/C9GC02475A

[CR34] Delic M, Valli M, Graf AB, Pfeffer M, Mattanovich D, Gasser B (2013) The secretory pathway: exploring yeast diversity. FEMS Microbiol Rev 37:872–914. 10.1111/1574-6976.12020 23480475 10.1111/1574-6976.12020

[CR35] Denks K, Vogt A, Sachelaru I, Petriman NA, Kudva R, Koch H-G (2014) The Sec translocon mediated protein transport in prokaryotes and eukaryotes. Mol Membr Biol 31:58–84. 10.3109/09687688.2014.907455 24762201 10.3109/09687688.2014.907455

[CR36] Ellgaard L, Helenius A (2003) Quality control in the endoplasmic reticulum. Nat Rev Mol Cell Biol 4:181–191. 10.1038/nrm1052 12612637 10.1038/nrm1052

[CR37] Eom GT, Lee SH, Song BK, Chung KW, Kim YW, Song JK (2013) High-level extracellular production and characterization of *Candida**antarctica* lipase B in *Pichia**pastoris*. J Biosci Bioeng 116:165–170. 10.1016/j.jbiosc.2013.02.016 23571105 10.1016/j.jbiosc.2013.02.016

[CR38] Eurwilaichitr L, Roytrakul S, Suprasongsin C, Manitchotpisit P, Panyim S (2002) Glutamic acid and alanine spacer is not necessary for removal of MFα-1 signal sequence fused to the human growth hormone produced from *Pichia**pastoris*. World J Microbiol Biotechnol 18:493–498. 10.1023/A:1016383326627

[CR39] Fabre E, Nicaud JM, Lopez MC, Gaillardin C (1991) Role of the proregion in the production and secretion of the Yarrowia lipolytica alkaline extracellular protease. J Biol Chem 266:3782–3790. 10.1016/S0021-9258(19)67863-4 1995632

[CR40] Feldheim D, Schekman R (1994) Sec72p contributes to the selective recognition of signal peptides by the secretory polypeptide translation complex. J Cell Biol 126:935–943. 10.1083/jcb.126.4.935 8051213 10.1083/jcb.126.4.935 PMC2120110

[CR41] Fitzgerald I, Glick BS (2014) Secretion of a foreign protein from budding yeasts is enhanced by cotranslational translocation and by suppression of vacuolar targeting. Microb Cell Fact 13:1–12. 10.1186/S12934-014-0125-0 25164324 10.1186/s12934-014-0125-0 PMC4176846

[CR42] Fitzgerald-Hayes M, Clarke L, Carbon J (1982) Nucleotide sequence comparisons and functional analysis of yeast centromere DNAs. Cell 29:235–244. 10.1016/0092-8674(82)90108-8 7049398 10.1016/0092-8674(82)90108-8

[CR43] Forte GMA, Pool MR, Stirling CJ (2011) N-terminal acetylation inhibits protein targeting to the endoplasmic reticulum. PLoS Biol 9:e1001073. 10.1371/journal.pbio.1001073 21655302 10.1371/journal.pbio.1001073 PMC3104963

[CR44] Freudl R (2018) Signal peptides for recombinant protein secretion in bacterial expression systems. Microb Cell Fact 17:52. 10.1186/s12934-018-0901-3 29598818 10.1186/s12934-018-0901-3 PMC5875014

[CR45] Fuller RS, Brakes A, Thorner J (1989) Yeast prohormone processing enzyme (KEX2 gene product) is a Ca2’-dependent serine protease. Proc Natl Acad Sci USA 86:1434–1438. 10.1073/pnas.86.5.1434 2646633 10.1073/pnas.86.5.1434 PMC286710

[CR46] Gellerfors P, Axelsson K, Helander A, Johansson S, Kennee L, Lindqvist S, Pavlu B, Skottner A, Fryklund L (1989) Isolation and characterization of a glycosylated form of human insulin-like growth factor I produced in *Saccharomyces**cerevisiae*. J Biol Chem 264:11444–11449. 10.1016/S0021-9258(18)60484-3 2500441

[CR47] Gellissen G, Janowicz ZA, Merckelbach A, Piontek M, Keup P, Weydemann U, Hollenberg CP, Strasser AWM (1991) Heterologous gene expression in *Hansenula**polymorpha*: efficient secretion of glucoamylase. Biotechniques 9:291–295. 10.1038/nbt0391-291 10.1038/nbt0391-291 1367303

[CR48] Gierasch LM, Kaiser T (1989) Signal sequences. Biochemistry 28:923–930. 10.1021/bi00429a001 2653440 10.1021/bi00429a001

[CR49] Gomes AMV, Carmo TS, Carvalho LS, Bahia FM, Parachin NS (2018) Comparison of yeasts as hosts for recombinant protein production. Microorganisms 6:38. 10.3390/microorganisms6020038 29710826 10.3390/microorganisms6020038 PMC6027275

[CR50] Gundry M, Vijg J (2012) Direct mutation analysis by high-throughput sequencing: from germline to low-abundant, somatic variants. Mutat Res - Fundam Mol Mech Mutagen 729:1–15. 10.1016/j.mrfmmm.2011.10.001 10.1016/j.mrfmmm.2011.10.001 PMC3237897 22016070

[CR51] Han M, Yu X (2015) Enhanced expression of heterologous proteins in yeast cells via the modification of N-glycosylation sites. Bioengineered 6:115–118. 10.1080/21655979.2015.1011031 25671496 10.1080/21655979.2015.1011031 PMC4601336

[CR52] Hann BC, Walter P (1991) The signal recognition particle in S. cerevisiae. Cell 67(1):131–144. 10.1016/0962-8924(92)90134-9 1655273 10.1016/0092-8674(91)90577-l

[CR53] Hou J, Tyo KEJ, Liu Z, Petranovic D, Nielsen J (2012) Metabolic engineering of recombinant protein secretion by *Saccharomyces**cerevisiae*. FEMS Yeast Res 12:491–510. 10.1111/j.1567-1364.2012.00810.x 22533807 10.1111/j.1567-1364.2012.00810.x

[CR54] Huang M, Wang G, Qin J, Petranovic D, Nielsen J (2018) Engineering the protein secretory pathway of *Saccharomyces**cerevisiae* enables improved protein production. Proc Natl Acad Sci USA. 10.1073/pnas.1809921115 30397111 10.1073/pnas.1809921115 PMC6255153

[CR55] Imai K, Nakai K (2020) Tools for the recognition of sorting signals and the prediction of subcellular localization of proteins from their amino acid sequences. Front Genet 11:607812. 10.3389/fgene.2020.607812 10.3389/fgene.2020.607812 PMC7723863 33324450

[CR56] Ito Y, Ishigami M, Hashiba N, Nakamura Y, Terai G, Hasunuma T, Ishii J, Kondo A (2022) Avoiding entry into intracellular protein degradation pathways by signal mutations increases protein secretion in *Pichia**pastoris*. Microb Biotechnol 15:2364–2378. 10.1111/1751-7915.14061 35656803 10.1111/1751-7915.14061 PMC9437885

[CR57] Jie CX, Wésolowski-Louvel M, Fukuhara H (1992) Glucose transport in the yeast *Kluyveromyces**lactis* II. Transcriptional regulation of the glucose transporter gene RAG1. Mol Gen Genomics 233:9–105. 10.1007/BF00587566 10.1007/BF00587566 1603079

[CR58] Jin Y, Yang N, Teng D, Hao Y, Mao R, Wang J (2023) Molecular modification of Kex2 P1’site enhances expression and druggability of fungal defensin. Antibiotics 12:786. 10.3390/antibiotics12040786 37107149 10.3390/antibiotics12040786 PMC10135057

[CR59] Johnson N, Powis K, High S (2013) Post-translational translocation into the endoplasmic reticulum. Biochim Biophys Acta 1833:2403–2409. 10.1016/J.BBAMCR.2012.12.008 23266354 10.1016/j.bbamcr.2012.12.008

[CR60] Jørgensen MU, Emr SD, Winther JR (1999) Ligand recognition and domain structure of Vps10p, a vacuolar protein sorting receptor in *Saccharomyces**cerevisiae*. Eur J Biochem 260:461–469. 10.1046/j.1432-1327.1999.00176.x 10095782 10.1046/j.1432-1327.1999.00176.x

[CR61] Joshi KK, Sahni G (2010) Molecular cloning, expression, purification and characterization of truncated forms of human plasminogen in Pichia pastoris expression system. Process Biochem 45:1251–1260. 10.1016/j.procbio.2010.04.014

[CR62] Julius D, Blair L, Brake A, Sprague G, Thorner J (1983) Yeast α factor is processed from a larger precursor polypeptide: the essential role of a membrane-bound dipeptidyl aminopeptidase. Cell 32:839–852. 10.1016/0092-8674(83)90070-3 6339075 10.1016/0092-8674(83)90070-3

[CR63] Julius D, Schekman R, Thorner J (1984) Glycosylation and processing of prepro-α-factor through the yeast secretory pathway. Cell 36:309–318. 10.1016/0092-8674(84)90224-1 6420074 10.1016/0092-8674(84)90224-1

[CR64] Kang Q, Zhang D (2020) Principle and potential applications of the non-classical protein secretory pathway in bacteria. Appl Microbiol Biotechnol 104:953–965. 10.1007/s00253-019-10285-4 31853566 10.1007/s00253-019-10285-4

[CR65] Karaoglan M, Yildiz H, Inan M (2014) Screening of signal sequences for extracellular production of *Aspergillus**niger* xylanase in *Pichia**pastoris*. Biochem Eng J 92:16–21. 10.1016/j.bej.2014.07.005

[CR66] Kim MJ, Park SL, Kim SH, Park HJ, Sung BH, Sohn JH, Bae JH (2023) Modulation of Kex2p cleavage site for in vitro processing of recombinant proteins produced *by Saccharomyces**cerevisiae*. J Microbiol Biotechnol 33:1513–1520. 10.4014/jmb.2306.06024 37482809 10.4014/jmb.2306.06024 PMC10699272

[CR67] Kjeldsen T, Brandt J, Andersen AS, Egel-Mitani M, Hach M, Pettersson AF, Vad K (1996) A removable spacer peptide in an α-factor-leader/insulin precursor fusion protein improves processing and concomitant yield of the insulin precursor in *Saccharomyces**cerevisiae*. Gene 170:107–112. 10.1016/0378-1119(95)00822-5 8621069 10.1016/0378-1119(95)00822-5

[CR68] Kjeldsen T, Pettersson AF, Hach M (1999) Secretory expression and characterization of insulin in *Pichia**pastoris*. Biotechnol Appl Biochem 29:79–86. 10.1111/j.1470-8744.1999.tb01151.x 9889087

[CR69] Klein T, Lange S, Wilhelm N, Bureik M, Yang TH, Heinzle E, Schneider K (2014) Overcoming the metabolic burden of protein secretion in *Schizosaccharomyces**pombe* - a quantitative approach using 13C-based metabolic flux analysis. Metab Eng 21:34–45. 10.1016/j.ymben.2013.11.001 24269998 10.1016/j.ymben.2013.11.001

[CR70] Krainer FW, Gerstmann MA, Darnhofer B, Birner-Gruenberger R, Glieder A (2016) Biotechnological advances towards an enhanced peroxidase production in *Pichia**pastoris*. J Biotechnol 233:181–189. 10.1016/j.jbiotec.2016.07.012 27432633 10.1016/j.jbiotec.2016.07.012

[CR71] Kuchler K, Rubartelli A, Holland B (1997) Unusual secretory pathways: from bacteria to man. 10.1007/978-3-662-22581-3

[CR72] Kurjan J, Herskowitz I (1982) Structure of a yeast pheromone gene (MFα): a putative α-factor precursor contains four tandem copies of mature α-factor. Cell 30:933–943. 10.1016/0092-8674(82)90419-3 6754095 10.1016/0092-8674(82)90298-7

[CR73] Lakkaraju AKK, Thankappan R, Mary C, Garrison JL, Taunton J, Strub K (2012) Efficient secretion of small proteins in mammalian cells relies on Sec62-dependent posttranslational translocation. Mol Biol Cell 23:2712–2722. 10.1091/mbc.E12-03-0228 22648169 10.1091/mbc.E12-03-0228 PMC3395660

[CR74] Li F, Chen Y, Qi Q, Wang Y, Yuan L, Huang M, Elsemman IE, Feizi A, Kerkhoven EJ, Nielsen J (2022) Improving recombinant protein production by yeast through genome-scale modeling using proteome constraints. Nat Commun 13:2969. 10.1038/s41467-022-30689-7 35624178 10.1038/s41467-022-30689-7 PMC9142503

[CR75] Lin-Cereghino GP, Stark CM, Kim D, Chang J, Shaheen N, Poerwanto H, Agari K, Moua P, Low LK, Tran N, Huang AD, Nattestad M, Oshiro KT, Chang JW, Chavan A, Tsai JW, Lin-Cereghino J (2013) The effect of α-mating factor secretion signal mutations on recombinant protein expression in *Pichia**pastoris*. Gene 519:311–317. 10.1016/j.gene.2013.01.062 23454485 10.1016/j.gene.2013.01.062 PMC3628533

[CR76] Liu C, Gong JS, Su C, Li H, Li H, Rao ZM, Xu ZH, Shi JS (2022) Pathway engineering facilitates efficient protein expression in *Pichia**pastoris*. Appl Microbiol Biotechnol 106:5893–5912. 10.1007/s00253-022-12139-y 36040488 10.1007/s00253-022-12139-y

[CR77] Loibl M, Wunderle L, Hutzler J, Schulz BL, Aebi M, Strahl S (2014) Protein O-mannosyltransferases associate with the translocon to modify translocating polypeptide chains. J Biol Chem 289:8599–8611. 10.1074/jbc.M113.543116 24519942 10.1074/jbc.M113.543116 PMC3961683

[CR78] Madzak C, Gaillardin C, Beckerich JM (2004) Heterologous protein expression and secretion in the non-conventional yeast *Yarrowia**lipolytica*: a review. J Biotechnol 109:63–81. 10.1016/j.jbiotec.2003.10.027 15063615 10.1016/j.jbiotec.2003.10.027

[CR79] Manfredi MA, Antunes AA, Jesus LDOP, Juliano MA, Juliano L, de Souza Judice WA (2016) Specificity characterization of the α-mating factor hormone by Kex2 protease. Biochimie 131:149–158. 10.1016/j.biochi.2016.10.003 27720750 10.1016/j.biochi.2016.10.003

[CR80] Mateljak I, Tron T, Alcalde M (2017) Evolved α-factor prepro-leaders for directed laccase evolution in *Saccharomyces**cerevisiae*. Microb Biotechnol 10:1830–1836. 10.1111/1751-7915.12838 28805314 10.1111/1751-7915.12838 PMC5658585

[CR81] Matlack KES, Misselwitz B, Plath K, Rapoport TA (1999) BiP acts as a molecular ratchet during posttranslational transport of Prepro- α -factor across the ER membrane. Cell 97:553–564. 10.1016/S0092-8674(00)80767-9 10367885 10.1016/s0092-8674(00)80767-9

[CR82] Matsuo Y, Akiyama N, Nakamura H, Yodoi J, Noda M, Kizaka-Kondoh S (2001) Identification of a novel thioredoxin-related transmembrane protein. J Biol Chem 276:10032–10038. 10.1074/jbc.M011037200 11152479 10.1074/jbc.M011037200

[CR83] Mattanovich D, Branduardi P, Dato L, Gasser B, Sauer M, Porro D (2012) Recombinant protein production in yeasts. Methods Mol Biol 824:329–358. 10.1007/978-1-61779-433-9_17 22160907 10.1007/978-1-61779-433-9_17

[CR84] Mehul B, Hughes CR (1997) Plasma membrane targetting, vesicular budding and release of galectin 3 from the cytoplasm of mammalian cells during secretion. J Cell Sci 110:1169–1178. 10.1242/jcs.110.10.1169 9191041 10.1242/jcs.110.10.1169

[CR85] Merkaš M, Pantelakis OI, Emmerstorfer-Augustin A (2025) Cloning of MFα secretion signal variants for protein secretion in *Pichia pastoris*. Garcia-Ortega X, Glieder A, Kovar K, Rieder L (eds) *Pichia pastoris: Methods and Protocols* 10.1007/978-1-0716-4779-0_16 41028467

[CR86] Miller JD, Tajima S, Lauffer L, Walter P (1995) The beta subunit of the signal recognition particle receptor is a transmembrane GTPase that anchors the alpha subunit, a peripheral membrane GTPase, to the endoplasmic reticulum membrane. J Cell Biol 128:273–282. 10.1083/jcb.128.3.273 7844142 10.1083/jcb.128.3.273 PMC2120348

[CR87] Mori A, Hara S, Sugahara T, Kojima T, Iwasaki Y, Kawarasaki Y, Sahara T, Ohgiya S, Nakano H (2015) Signal peptide optimization tool for the secretion of recombinant protein from *Saccharomyces**cerevisiae*. J Biosci Bioeng 120:518–525. 10.1016/j.jbiosc.2015.03.003 25912446 10.1016/j.jbiosc.2015.03.003

[CR88] Näätsaari L, Mistlberger B, Ruth C, Hajek T, Hartner FS, Glieder A (2012) Deletion of the *Pichia**pastoris* KU70 homologue facilitates platform strain generation for gene expression and synthetic biology. PLoS ONE 7:e39720. 10.1371/journal.pone.0039720 22768112 10.1371/journal.pone.0039720 PMC3387205

[CR89] Neiers F, Belloir C, Poirier N, Naumer C, Krohn M, Briand L (2021) Comparison of different signal peptides for the efficient secretion of the sweet-tasting plant protein brazzein in *Pichia**pastoris*. Life 11:1–12. 10.3390/life11010046 10.3390/life11010046 PMC7828362 33450886

[CR90] Ng DTW, Brown JD, Walter P (1996) Signal sequences specify the targeting route to the endoplasmic reticulum membrane. J Cell Biol 134:267–278. 10.1083/jcb.134.2.269 10.1083/jcb.134.2.269 PMC2120870 8707814

[CR91] Nickel W, Rabouille C (2009) Mechanisms of regulated unconventional protein secretion. Nat Rev Mol Cell Biol 10:148–155. 10.1038/nrm2617 19122676 10.1038/nrm2617

[CR92] Nickel W, Seedorf M (2008) Unconventional mechanisms of protein transport to the cell surface of eukaryotic cells. Annu Rev Cell Dev Biol 24:287–308. 10.1146/annurev.cellbio.24.110707.175320 18590485 10.1146/annurev.cellbio.24.110707.175320

[CR93] Nielsen H, Tsirigos KD, Brunak S, von Heijne G (2019) A brief history of protein sorting prediction. Protein J 38:200–216. 10.1007/s10930-019-09838-3 31119599 10.1007/s10930-019-09838-3 PMC6589146

[CR94] Nogueira ES, Schleier T, Dürrenberger M, Ballmer-Hofer K, Ward TR, Jaussi R (2014) High-level secretion of recombinant full-length streptavidin in Pichia pastoris and its application to enantioselective catalysis. Protein Expr Purif 93:54–62. 10.1016/j.pep.2013.10.015 24184946 10.1016/j.pep.2013.10.015

[CR95] Obst U, Lu TK, Sieber V (2017) A modular toolkit for generating *Pichia**pastoris* secretion libraries. ACS Synth Biol 6:1016–1025. 10.1021/acssynbio.6b00337 28252957 10.1021/acssynbio.6b00337

[CR96] O’Riordan NM, Jurić V, O’Neill SK, Roche AP, Young PW (2024) A yeast modular cloning (MoClo) toolkit expansion for optimization of heterologous protein secretion and surface display in Saccharomyces cerevisiae. ACS Synth Biol. 13(4):1246–1258. 10.1021/acssynbio.3c00743 38483353 10.1021/acssynbio.3c00743 PMC11036508

[CR97] Owji H, Nezafat N, Negahdaripour M, Hajiebrahimi A, Ghasemi Y (2018) A comprehensive review of signal peptides: structure, roles, and applications. Eur J Cell Biol 97:422–441. 10.1016/j.ejcb.2018.06.003 29958716 10.1016/j.ejcb.2018.06.003

[CR98] Plath K, Rapoport TA (2000) Spontaneous release of cytosolic proteins from posttranslational substrates before their transport into the endoplasmic reticulum. J Cell Biol 151:167–178. 10.1083/jcb.151.1.167 11018062 10.1083/jcb.151.1.167 PMC2189806

[CR99] Plath K, Mothes W, Wilkinson BM, Stirling CJ, Rapoport TA (1998) Signal sequence recognition in posttranslational protein transport across the yeast ER membrane. Cell 94:795–807. 10.1016/S0092-8674(00)81738-9 9753326 10.1016/s0092-8674(00)81738-9

[CR100] Plath K, Wilkinson BM, Stirling CJ, Rapoport TA (2004) Interactions between sec complex and prepro-factor during posttranslational protein transport into the endoplasmic reticulum. Mol Biol Cell 15:1–10. 10.1091/mbc.E03-06 14617809 10.1091/mbc.E03-06-0390 PMC307522

[CR101] Pobre KFR, Poet GJ, Hendershot LM (2019) The endoplasmic reticulum (ER) chaperone BiP is a master regulator of ER functions: getting by with a little help from ERdj friends. J Biol Chem 294:2098–2108. 10.1074/jbc.REV118.002804 30563838 10.1074/jbc.REV118.002804 PMC6369273

[CR102] Poirier N, Roudnitzky N, Brockhoff A, Belloir C, Maison M, Thomas-Danguin T, Meyerhof W, Briand L (2012) Efficient production and characterization of the sweet-tasting brazzein secreted by the *yeast Pichia**pastoris*. J Agric Food Chem 60:9807–9814. 10.1021/jf301600m 22958103 10.1021/jf301600m

[CR103] Pool MR (2022) Targeting of proteins for translocation at the endoplasmic reticulum. Int J Mol Sci 23:3773. 10.3390/ijms23073773 35409131 10.3390/ijms23073773 PMC8998515

[CR104] Porro D, Sauer M, Branduardi P, Mattanovich D (2005) Recombinant protein production in yeasts. Mol Biotechnol 31:245–259. 10.1007/978-1-61779-433-9_17 16230775 10.1385/MB:31:3:245

[CR105] Prabhu AA, Bharali B, Singh AK, Allaka M, Sukumar P, Veeranki VD (2018) Engineering folding mechanism through Hsp70 and Hsp40 chaperones for enhancing the production of recombinant human interferon gamma (hIFN-γ) in *Pichia**pastoris* cell factory. Chem Eng Sci 181:58–67. 10.1016/j.ces.2018.02.003

[CR106] Puxbaum V, Mattanovich D, Gasser B (2015) Quo vadis? The challenges of recombinant protein folding and secretion in *Pichia**pastoris*. Appl Microbiol Biotechnol 99:2925–2938. 10.1007/s00253-015-6470-z 25722021 10.1007/s00253-015-6470-z

[CR107] Rabouille C (2017) Pathways of unconventional protein secretion. Trends Cell Biol 27:230–240. 10.1016/j.tcb.2016.11.007 27989656 10.1016/j.tcb.2016.11.007

[CR108] Radanović T, Ernst R (2021) The unfolded protein response as a guardian of the secretory pathway. Cells 10:2965. 10.3390/cells10112965 34831188 10.3390/cells10112965 PMC8616143

[CR109] Rakestraw JA, Sazinsky SL, Piatesi A, Antipov E, Wittrup KD (2009) Directed evolution of a secretory leader for the improved expression of heterologous proteins and full-length antibodies in S. cerevisiae. Biotechnol Bioeng 103:1192. 10.1002/BIT.22338 19459139 10.1002/bit.22338 PMC2847895

[CR110] Raschmanová H, Weninger A, Knejzlík Z, Melzoch K, Kovar K (2021) Engineering of the unfolded protein response pathway in *Pichia**pastoris*: enhancing production of secreted recombinant proteins. Appl Microbiol Biotechnol 105:4397–4414. 10.1007/s00253-021-11336-5 34037840 10.1007/s00253-021-11336-5 PMC8195892

[CR111] Rieder L, Ebner K, Glieder A, Sørlie M (2021) Novel molecular biological tools for the efficient expression of fungal lytic polysaccharide monooxygenases in Pichia pastoris. Biotechnol Biofuels 14:122. 10.1186/s13068-021-01971-5 34044872 10.1186/s13068-021-01971-5 PMC8161572

[CR112] Robinson PJ, Bulleid NJ (2020) Mechanisms of disulfide bond formation in nascent polypeptides entering the secretory pathway. Cells 9:1994. 10.3390/cells9091994 10.3390/cells9091994 PMC7565403 32872499

[CR113] Rockwell NC, Krysan DJ, Komiyama T, Fuller RS (2002) Precursor processing by Kex2/Furin proteases. Chem Rev 102:4525–4548. 10.1021/cr010168i 12475200 10.1021/cr010168i

[CR114] Roh JY, Koo BC, Kwon MS, Kim M, Kim NH, Kim T (2013) Modification of enhanced green fluorescent protein for secretion out of cells. Biotechnol Bioprocess Eng 18:1135–1141. 10.1007/s12257-013-0333-1

[CR115] Roongsawang N, Puseenam A, Kitikhun S, Sae-Tang K, Harnpicharnchai P, Ohashi T, Fujiyama K, Tirasophon W, Tanapongpipat S (2016) A novel potential signal peptide sequence and overexpression of ER-resident chaperones enhance heterologous protein secretion in thermotolerant methylotrophic yeast Ogataea thermomethanolica. Appl Biochem Biotechnol 178:710–724. 10.1007/s12010-015-1904-8 26519344 10.1007/s12010-015-1904-8

[CR116] Sakai Y, Akiyama M, Kondoh H, Shibano Y, Kato N (1996) High-level secretion of fungal glucoamylase using the *Candida**boidinii* gene expression system. Biochim Biophys Acta (BBA) - Gene Struct Expr 1308:81–87. 10.1016/0167-4781(96)00075-9 10.1016/0167-4781(96)00075-9 8765754

[CR117] Schatz G, Dobberstein B (1996) Common principles of protein translocation across membranes. Science 271:1519–1526. 10.1126/science.271.5255.1519 8599107 10.1126/science.271.5255.1519

[CR118] Shen Q, Zhou XT, Guo Q, Xue YZ, Xue YP, Zheng YG (2022) Potential of the signal peptide derived from the PAS_chr3_0030 gene product for secretory expression of valuable enzymes in *Pichia**pastoris*. Appl Environ Microbiol 88:e00296-22. 10.1128/aem.00296-22 35435711 10.1128/aem.00296-22 PMC9088389

[CR119] Siegel RS, Buckholz RG, Thill GP, Wondrack LM (1990) Production of epidermal growth factor in methylotrophic yeast cells. WO Patent 90/10697

[CR120] Simons JF, Ferro-Novick S, Rose MD, Helenius A (1995) BiP/Kar2p serves as a molecular chaperone during carboxypeptidase Y folding in yeast. J Cell Biol 130:41–49. 10.1083/jcb.130.1.41 7790376 10.1083/jcb.130.1.41 PMC2120506

[CR121] Sosa-Carrillo S, Galez H, Napolitano S, Bertaux F, Batt G (2023) Maximizing protein production by keeping cells at optimal secretory stress levels using real-time control approaches. Nat Commun 14:3028. 10.1038/s41467-023-38807-9 37231013 10.1038/s41467-023-38807-9 PMC10212943

[CR122] Southgate VJ, Steyn AJ, Pretorius IS, Van Vuuren HJ (1993) Expression and secretion of *Bacillus**amyloliquefaciens* alpha-amylase by using the yeast pheromone alpha-factor promoter and leader sequence in Saccharomyces cerevisiae. Appl Environ Microbiol 59:1253–1258. 10.1128/aem.59.4.1253-1258.1993 8476297 10.1128/aem.59.4.1253-1258.1993 PMC202271

[CR123] Spohner SC, Schaum V, Quitmann H, Czermak P (2016) *Kluyveromyces**lactis*: an emerging tool in biotechnology. J Biotechnol 222:104–116. 10.1016/j.jbiotec.2016.02.023 26912289 10.1016/j.jbiotec.2016.02.023

[CR124] Srikant S, Gaudet R, Murray AW (2021) Beyond the reach of homology: successive computational filters find yeast pheromone genes. bioRxiv 2021:2021–09. 10.1101/2021.09.28.462209

[CR125] Sun Z, Brodsky JL (2019) Protein quality control in the secretory pathway. J Cell Biol 218:3171–3187. 10.1083/jcb.201906047 10.1083/jcb.201906047 PMC6781448 31537714

[CR126] Suzuki M (2020) Structural and functional analyses of organic molecules regulating biomineralization. Biosci Biotechnol Biochem 84:1529–1540. 10.1080/09168451.2020.1762068 32434433 10.1080/09168451.2020.1762068

[CR127] Tan NS, Ho B, Ding JL (2002) Engineering a novel secretion signal for cross-host recombinant protein expression. Protein Eng 15:337–345. 10.1093/protein/15.4.337 11983935 10.1093/protein/15.4.337

[CR128] Teufel F, Almagro Armenteros JJ, Johansen AR, Gíslason MH, Pihl SI, Tsirigos KD, Winther O, Brunak S, von Heijne G, Nielsen H (2022) SignalP 6.0 predicts all five types of signal peptides using protein language models. Nat Biotechnol 40:1023–1025. 10.1038/s41587-021-01156-3 34980915 10.1038/s41587-021-01156-3 PMC9287161

[CR129] Teufl M, Zajc CU, Traxlmayr MW (2022) Engineering strategies to overcome the stability–function trade-off in proteins. ACS Synth Biol 11:1030–1039. 10.1021/acssynbio.1c00512 35258287 10.1021/acssynbio.1c00512 PMC8938945

[CR130] Tschopp JF, Sverlow G, Kosson R, Craig W, Grinna L (1987) High-level secretion of glycosylated invertase in the methylotrophic yeast, *Pichia**pastoris*. Bio/technology 5:1305–1308. 10.1038/nbt1287-1305

[CR131] Utami N, Nurdiani N, Hariyatun H, Putro EW, Patria FP, Kusharyoto W (2023) Full-length versus truncated α-factor secretory signal sequences for expression of recombinant human insulin precursor in yeast *Pichia**pastoris*: a comparison. J Genet Eng Biotechnol 21:67. 10.1186/s43141-023-00521-w 37212962 10.1186/s43141-023-00521-w PMC10203085

[CR132] Vadhana AKP, Samuel P, Berin RM, Krishna J, Kamatchi K, Meenakshisundaram S (2013) Improved secretion of *Candida**antarctica* lipase B with its native signal peptide in *Pichia**pastoris*. Enzyme Microb Technol 52:177–183. 10.1016/j.enzmictec.2013.01.001 23410929 10.1016/j.enzmictec.2013.01.001

[CR133] Vassileva A, Chugh DA, Swaminathan S, Khanna N (2001) Expression of hepatitis B surface antigen in the methylotrophic yeast *Pichia**pastoris* using the GAP promoter. J Biotechnol 88:21–35. 10.1016/S0168-1656(01)00254-1 11377762 10.1016/s0168-1656(01)00254-1

[CR134] Vogl T, Sturmberger L, Kickenweiz T, Wasmayer R, Schmid C, Hatzl A-M, Gerstmann MA et al (2016) A toolbox of diverse promoters related to methanol utilization: functionally verified parts for heterologous pathway expression in *Pichia**pastoris*. ACS Synth Biol 5:172–186. 10.1021/acssynbio.5b00199 26592304 10.1021/acssynbio.5b00199

[CR135] Vogl T, Elgin Fischer J, Hyden P, Wasmayer R, Sturmberger L, Glieder A (2020) Orthologous promoters from related methylotrophic yeasts surpass expression of endogenous promoters of *Pichia**pastoris*. AMB Express 10:1–9. 10.1186/s13568-020-00972-1 10.1186/s13568-020-00972-1 PMC7042429 32100120

[CR136] von Heijne G (1984) How signal sequences maintain cleavage specificity. J Mol Biol 173:243–251. 10.1016/0022-2836(84)90192-X 6423828 10.1016/0022-2836(84)90192-x

[CR137] von Heijne G (1990) The signal peptide. J Membr Biol 115:195–201. 10.1007/BF01868635 2197415 10.1007/BF01868635

[CR138] Voos W, Stevens TH (1998) Retrieval of resident late-Golgi membrane proteins from the prevacuolar compartment of *Saccharomyces**cerevisiae* is dependent on the function of Grd19p. J Cell Biol 140:577–590. 10.1083/jcb.140.3.577 9456318 10.1083/jcb.140.3.577 PMC2140161

[CR139] Wang H, Lu F, Sun Y, Du L (2004) Heterologous expression of lignin peroxidase of *Phanerochaete**chrysosporium* in *Pichia**methanolica*. Biotechnol Lett 26:1569–1573. 10.1023/B:BIOL.0000045130.11091.57 15604798 10.1023/B:BILE.0000045654.66689.b4

[CR140] Wang J, Li J, Hou Y, Dai W, Xie R, Marquez-Lago TT, Leier A, Zhou T, Torres V, Hay I, Stubenrauch C, Zhang Y, Song J, Lithgow T (2021) BastionHub: a universal platform for integrating and analyzing substrates secreted by Gram-negative bacteria. Nucleic Acids Res 49:D651–D659. 10.1093/nar/gkaa899 33084862 10.1093/nar/gkaa899 PMC7778982

[CR141] Wartmann T, Boer E, Pico AH, Sieber H, Bartelsen O, Gellissen G, Kunze G (2002) High-level production and secretion of recombinant proteins by the dimorphic yeast *Arxula**adeninivorans*. FEMS Yeast Res 2:363–369. 10.1111/j.1567-1364.2002.tb00105.x 12702286 10.1016/S1567-1356(02)00086-7

[CR142] Werten MW, de Wolf FA (2005) Reduced proteolysis of secreted gelatin and Yps1-mediated α-factor leader processing in a *Pichia**pastoris* kex2 disruptant. Appl Environ Microbiol 71:2310–2317. 10.1128/AEM.71.5.2310-2317.2005 15870316 10.1128/AEM.71.5.2310-2317.2005 PMC1087524

[CR143] Weydemann U, Keup P, Piontek M, Strasser AWM, Schweden J, Gellissen G, Janowicz ZA (1995) High-level secretion of hirudin by *Hansenula**polymorpha* -authentic processing of three different preprohirudins. Appl Microbiol Biotechnol 44:377–385. 10.1007/BF00169932 8597538 10.1007/BF00169932

[CR144] Wigginton JE, Abecasis GR (2005) PEDSTATS: descriptive statistics, graphics and quality assessment for gene mapping data. Bioinformatics 21:3445–3447. 10.1093/bioinformatics/bti529 15947021 10.1093/bioinformatics/bti529

[CR145] Willer M, Forte GMA, Stirling CJ (2008) Sec61p is required for ERAD-L: genetic dissection of the translocation and ERAD-L functions of Sec61P using novel derivatives of CPY. J Biol Chem 283:33883–33888. 10.1074/jbc.M803054200 18819915 10.1074/jbc.M803054200 PMC2590686

[CR146] Xiao D, Li X, Zhang Y, Wang F (2023) Efficient expression of *Candida**antarctica* Lipase B in *Pichia**pastoris* and its application in biodiesel production. Appl Biochem Biotechnol 195:5933–5949. 10.1007/s12010-023-04374-4 36723721 10.1007/s12010-023-04374-4

[CR147] Xie YF, Chen H, Huang BR (2007) Expression, purification and characterization of human IFN-λ1 in *Pichia**pastoris*. J Biotechnol 129:472–480. 10.1016/j.jbiotec.2007.01.018 17349709 10.1016/j.jbiotec.2007.01.018

[CR148] Xiong AS, Yao QH, Peng RH, Han PL, Cheng ZM, Li Y (2005) High level expression of a recombinant acid phytase gene in *Pichia**pastoris*. J Appl Microbiol 98:418–428. 10.1111/j.1365-2672.2004.02476.x 15659196 10.1111/j.1365-2672.2004.02476.x

[CR149] Xue S, Liu X, Pan Y, Xiao C, Feng Y, Zheng L, Zhao M, Huang M (2023) Comprehensive analysis of signal peptides in Saccharomyces cerevisiae reveals features for efficient secretion. Adv Sci (Weinh) 10(2):e2203433. 10.1002/advs.202203433 36478443 10.1002/advs.202203433 PMC9839866

[CR150] Yang S, Kuang Y, Li H, Liu Y, Hui X, Li P, Jiang Z, Zhou Y, Wang Y, Xu A, Li S, Liu P, Wu D (2013) Enhanced production of recombinant secretory proteins in *Pichia**pastoris* by Optimizing Kex2 P1’ site. PLoS ONE 8:e75347. 10.1371/journal.pone.0075347 24069404 10.1371/journal.pone.0075347 PMC3777899

[CR151] Zhang AL, Luo JX, Zhang TY, Pan YW, Tan YH, Fu CY, Tu FZ (2009) Recent advances on the GAP promoter derived expression system of *Pichia**pastoris*. Mol Biol Rep 36:1611–1619. 10.1007/s11033-008-9359-4 18781398 10.1007/s11033-008-9359-4

[CR152] Zhao HL, He Q, Xue C, Sun B, Yao XQ, Liu ZM (2009) Secretory expression of glycosylated and aglycosylated mutein of onconase from *Pichia**pastoris* using different secretion signals and their purification and characterization. FEMS Yeast Res 9:591–599. 10.1111/j.1567-1364.2009.00498.x 19416372 10.1111/j.1567-1364.2009.00498.x

[CR153] Zhu X, Li M, Zhu R, Xin Y, Guo Z, Gu Z, Zhang L, Guo Z (2023) Up front unfolded protein response combined with early protein secretion pathway engineering in *Yarrowia**lipolytica* to attenuate er stress caused by enzyme overproduction. J Mol Sci 2:16426. 10.3390/ijms242216426 10.3390/ijms242216426 PMC10670989 38003616

[CR154] Zimmermann R, Eyrisch S, Ahmad M, Helms V (2011) Protein translocation across the ER membrane. Biochim Biophys Acta Biomembr 1808:912–924. 10.1016/j.bbamem.2010.06.015 10.1016/j.bbamem.2010.06.015 20599535

[CR155] Zou C, Lu L, Wang S, Zhang C, Chen X, Lin Y, Huang Y (2022) The α-mating factor secretion signals and endogenous signal peptides for recombinant protein secretion in *Komagataella**phaffii*. Biotechnol Biofuels Bioprod 15:140. 10.1186/s13068-022-02243-6 36527112 10.1186/s13068-022-02243-6 PMC9756452

[CR156] Zsebo KM, Lu HS, Fieschko JC, Goldstein L, Davis J, Duker K, Suggs SV, Lai PH, Bitter GA (1986) Protein secretion from *Saccharomyces**cerevisiae* directed by the prepro-alpha-factor leader region. J Biol Chem 261:5858–5865. 10.1016/S0021-9258(17)38462-4 3009432

